# Age and cognitive skills: Use it or lose it

**DOI:** 10.1126/sciadv.ads1560

**Published:** 2025-03-05

**Authors:** Eric A. Hanushek, Lavinia Kinne, Frauke Witthöft, Ludger Woessmann

**Affiliations:** ^1^ Hoover Institution, Stanford University, Stanford, CA 94305, USA.; ^2^ CESifo, Munich 81679, Germany.; ^3^ IZA, Bonn 53113, Germany.; ^4^ NBER, Cambridge, MA 02138, USA.; ^5^ DIW Berlin, Berlin 10117, Germany.; ^6^ Berlin School of Economics, Berlin 10099, Germany.; ^7^ University of Potsdam, Potsdam 14469, Germany.; ^8^ ifo Institute at the University of Munich, Munich 81679, Germany.; ^9^ University of Munich, Munich 80539, Germany.

## Abstract

Cross-sectional age-skill profiles suggest that cognitive skills start declining by age 30 if not earlier. If accurate, such age-driven skill losses pose a major threat to the human capital of societies with rapidly aging populations. We estimate actual age-skill profiles from individual changes in literacy and numeracy skills at different ages. We use the unique German longitudinal component of the Programme of the International Assessment of Adult Competencies (PIAAC-L) that retested a large representative sample of adults after 3.5 years. Our empirical approach separates age from cohort effects and corrects for measurement error from reversion to the mean. Two main results emerge. First, average skills increase strongly into the forties before decreasing slightly in literacy and more strongly in numeracy. Second, skills decline at older ages only for those with below-average skill usage. White-collar and higher-educated workers with above-average usage show increasing skills even beyond their forties. Women have larger skill losses at older age, particularly in numeracy.

## Teaser

Cognitive skills do not decline with age for those who use math and reading throughout their life.

## INTRODUCTION

The commonly accepted conclusion that cognitive skills decline with age starting rather early in adult life (*1*) has increasingly important potential economic implications. Cognitive skills measured by literacy and numeracy are closely related to individual earnings (*2*–*4*) and national growth rates (*5*, *6*), implying that the steady and marked changes in the age composition of societies (*7*) might directly affect the economic well-being of nations. However, this assumed skill pattern has largely come from cross-sectional data that necessarily incorporate not only aging patterns but also cohort differences in skills. If the negative age pattern is simply due to conflating age and cohort effects, the economic concerns are considerably lessened. Recently developed individual longitudinal skill tests for a representative population of adults allow us to provide evidence on actual changes in skills with age and on their relationship with adult skill usage.

We study the inevitability of age-driven skill decline using exceptional longitudinal data for the German adult population. Administered by the Organization for Economic Co-operation and Development (OECD), the Programme for the International Assessment of Adult Competencies (PIAAC) tested literacy and numeracy skills that are relevant for participation in social life and work, and surveyed economic and social conditions for random samples of the population aged 16 to 65 in 39 countries (*8*). Germany, unique among all participating countries, created a panel of participants from the PIAAC sample who were re-surveyed and retested 3.5 years after the original survey.

We use the panel dimension of the data to estimate credible average age-skill profiles for the adult population. Observing actual changes in adult skills over the full age spectrum allows us to break the confounding of age and cohort patterns that has been ubiquitous in general cross-sectional analyses of adult skills. We estimate the average annualized marginal changes in skills for each age and concatenate them across individuals of different ages to derive full age-skill profiles. However, the panel data introduce new methodological problems that must be addressed before the analysis of age-skill patterns.

Creating reliable age-skill patterns based on individual aging requires addressing the bias from measurement errors that inevitably accompany testing of skills over time. Observations of test scores include a combination of true scores and measurement errors, leading to systematic errors when looking at individual changes with age. Intuitively, low observed test scores are more likely to include negative errors. When we observe another assessment for an initially low-scoring individual, the measurement error is unlikely to be as negative as the first time, implying that the change in true test scores is biased upward for low-scoring individuals. For initially high-scoring individuals, the opposite will be true. This reversion to the mean will bias the overall age-skill relationship when skills vary by age. Thus, we correct the observed change in test scores throughout for reversion to the mean (*9*) to obtain error-adjusted age-skill patterns.

We find that average (error-corrected) skills increase substantially into the forties for both literacy and numeracy. Subsequently, average skills decline slightly in literacy and strongly in numeracy. The averages, however, mask important heterogeneity.

With the appropriately adjusted age-skill profiles, we can further investigate the role of skill usage. Previous analyses have considered whether individual background or occupations influence the evolution of age-skill patterns. These investigations of heterogeneity in age patterns are generally motivated by assumed differences in skill usage across groups, but data on skill usage have not typically been available. Because the background data from the PIAAC survey provide information on the detailed nature and frequency of participants’ skill usage at work and at home, we are able to explore the demographic aging patterns in greater depth.

Individuals with above-average skill usage at work and home on average never face a skill decline (at least until the limit of our data at age 65). Consistent with the assumptions of prior studies, usage interacts closely with a variety of background characteristics. Thus, both literacy and numeracy skills keep increasing for white-collar and tertiary-educated workers in the second half of their working life if they have above-median skill usage, but not if skill usage is below the median. Skill evolution also varies by gender: Women show steeper skill decline at older ages, especially in numeracy.

The primary contribution of our analysis to the literature on cognitive aging is the development of rich age-skill profiles for literacy and numeracy skills, skills which have been shown to have economic payoffs in the labor market, using longitudinal variation in a large representative sample. The data allow us to go beyond averages to consider important dimensions of heterogeneity and to describe the connection of profile differences with individual behavior and background. The studies closest to our analysis use different large-scale representative surveys with literacy and numeracy skills (*1*, *10*), but these do not track actual skill changes of individuals over time. A parallel set of psychological and neuroscience studies offer related estimates of age patterns and their sources, albeit for different dimensions of skills and for nonrepresentative samples (*11*–*16*), but evidence on their relationship to the economic outcomes that motivate our work is lacking.

## RESULTS

### Average age-skill profiles

**Fig. 1.**
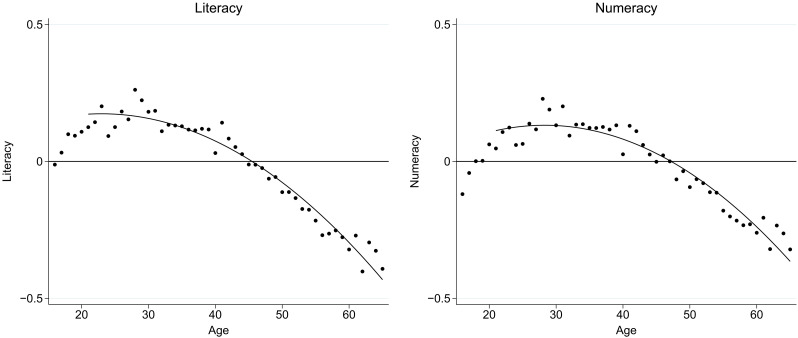
Cross-sectional age-skill profiles (OECD countries). Cross-sectional association between age and skills in PIAAC (2012). Dots: Average skills by age. Line: Quadratic fit (estimated over 21 to 65 age range). Skills measured in SD units. Sample: 25 OECD countries with continuous age data; full population, ages 16 to 65, weighted by sampling weights (*N* = 147,667). Data source: PIAAC.

The starting point for our analysis comes from the cross-sectional picture of how cognitive skills vary with age. Figure 1 depicts adult literacy and numeracy skills by age in the PIAAC test for representative population samples of all participating OECD countries [see also (*8*, *17*)]. In the cross section, average literacy and numeracy scores start declining in the late twenties to early thirties. This pattern is duplicated qualitatively for Germany, our analysis country, where average literacy scores steadily fall from age 20, while numeracy scores rise slightly before beginning to decline in the late thirties (fig. S1).

However, it is difficult to interpret these cross-sectional patterns because they conflate age and cohort effects. Individuals across different ages also come from different cohorts and have thus experienced different histories of skill determinants, implying that these charts fail to describe the true patterns of the age-skill relationship over time for any individual. As a result, they also cannot reliably be used to understand the factors that feed into skill changes by age without introducing strong assumptions about the nature of cohort and individual time patterns.

**Fig. 2.**
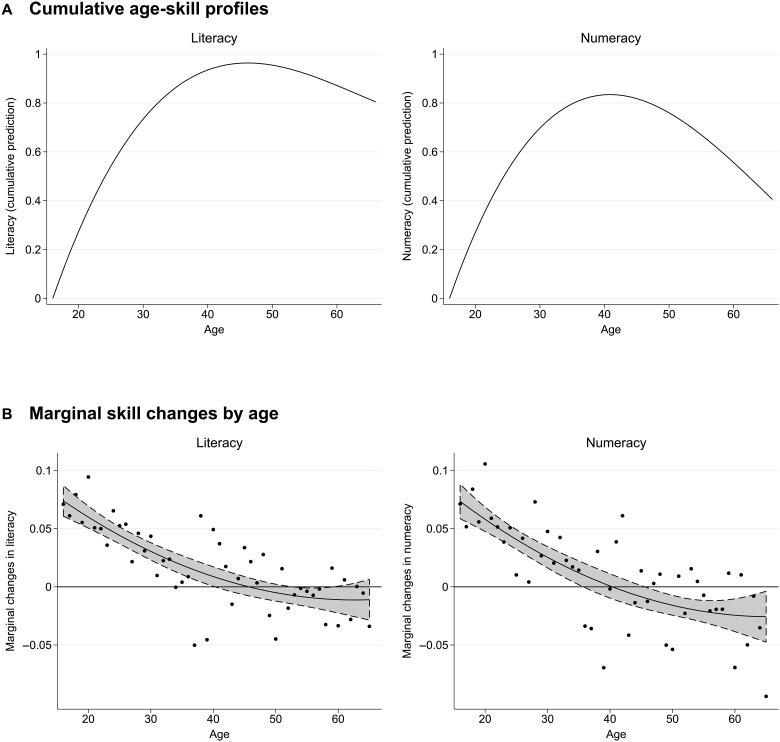
Longitudinal age-skill profiles. (**A**) Cumulative depiction of the predicted marginal change in skills at each age. (**B**) Marginal annualized change in skills between the two waves by age, adjusted for reversion to the mean. Dots: Average individual marginal annualized change in skills by age. Line: Quadratic fit. Gray area: 95% confidence interval. Skills measured in SD units. Sample: Full population, ages 16 to 65, weighted by sampling weights (*N* = 3263). Data source: PIAAC-L.

The changes in observed numeracy and literacy skills for individuals over time show a very different pattern than suggested by the cross-sectional data. Figure 2B depicts the annualized marginal changes in scores with age for individuals at each age for the full German population sample (3263 observations). The analysis corrects for reversion to the mean, and it includes a quadratic fit and 95% confidence intervals. The quadratic prediction indicates increases in skills for individuals up to age 45 in literacy and up to age 40 in numeracy. Skill changes turn negative beyond these ages, with notably stronger declines for numeracy than for literacy. In both subjects, the marginal change in skills declines steadily with age. Initially, this decline is close to linear but ultimately gets flatter.

Cumulative age-skill profiles that concatenate these marginal changes are displayed in Fig. 2A. Literacy skills increase strongly in the twenties and thirties and tend to stabilize and flatten starting in the late thirties. The peak is at age 46, but subsequent declines are limited. Numeracy skills also increase strongly at young ages but peak earlier, at age 41, and decline substantially at later ages, although not below the levels observed in the early twenties. Skill changes with age and age-skill profiles look quite similar when estimated just for individuals aged 25 and older (fig. S2), implying that the full-sample results are not driven by patterns in the age group 16 to 24 where many individuals are still in education.

The difference in age patterns across subjects may seem unexpected, not least because achievement in the two subjects is strongly positively correlated in the cross-section (correlation of 0.86). However, it is consistent with differences in aggregate patterns of skill usage by subject (fig. S3). Still, these average profiles hide important behavior-driven variations, the subject of the next section.

### Age-skill patterns by skill usage and background

Previous studies frequently suggest a considerable heterogeneity in age-skill patterns across individuals with different backgrounds that is motivated by assumed differences in cognitive activities. We extend this line of inquiry by explicitly considering how skill trajectories relate to individuals’ usage of skills. For these analyses, we focus on the sample of employed workers (2497 observations) for whom we can observe occupations and skill usage patterns at work and at home. The overall age-skill profiles for the employed sample (fig. S4) look very similar to the one for the full population (Fig. 2).

#### Heterogeneity by usage

When we separate age-skill profiles by the frequency of skill usage, we get notably different pictures. We repeat our prior analysis for samples divided at the median of the aggregate measure of skill usage at work and at home derived from the initial survey. In particular, we create an index based on the frequency of reported activities related to reading and math at work and in everyday life. Examples include “calculating prices, costs, or budgets” for math or “reading letters, memos, or e-mails” for reading, and frequencies range from “never” to “every day.”

**Fig. 3.**
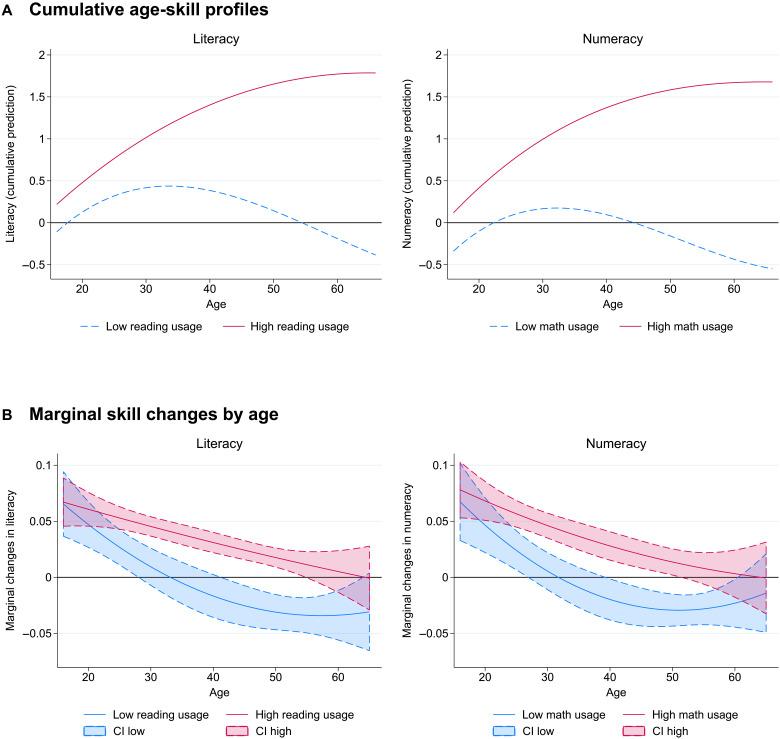
Age-skill profiles by skill usage. (**A**) Cumulative depiction of the predicted marginal change in skills at each age. (**B**) Quadratic fit [with 95% confidence interval (CI)] of marginal annualized change in skills between the two waves by age, adjusted for reversion to the mean. Skills measured in SD units. Sample split by median of skill usage at work and at home. Sample: Employed workers, ages 16 to 65, weighted by sampling weights (*N* = 2497). Data source: PIAAC-L.

Notably, those with above-median usage of each respective skill on average never show a decline in skills in the observed age range (Fig. 3). Their skills increase steeply into the fifties and then flatten out, with no indication of average decline. By contrast, for those with below-median usage, skill decline begins in their mid-thirties. In contrast to the aggregate pattern, the usage-specific patterns are quite similar for the two skill dimensions. Again, the pattern is qualitatively unchanged when disregarding individuals younger than 25 in the analysis (fig. S5). These results are consistent with skill usage playing a leading role in determining whether skills are gained, retained, or lost over time.

Results look qualitatively similar when considering skill usage at work and at home separately (figs. S6 and S7). Skill usage at home is observed not just in the sample of employed, but in the full population sample; full-population results look very similar as well (fig. S8).

The findings about age-skill patterns for the low- and high-usage groups in Fig. 3 also vividly demonstrate the importance of adjustment for reversion to the mean. The key differences in age-skill patterns with usage are distorted when looking at the raw, unadjusted skill data. The high-usage group has above-the-mean average test performance, implying that changes will be biased downward by the error pattern if uncorrected. With uncorrected data, the high-usage group appears to have lower growth in literacy skills with age and to lose numeracy skills after age 40 (fig. S9). On the other hand, for both skill categories, the low-usage group appears to show much lower declines in skills at older age, the kind of positive bias that reversion to the mean causes for observations with initially low scores. Looking at these patterns with unadjusted data would thus change the conclusions about age-skill patterns.

#### Heterogeneity by background characteristics

Prior analyses have focused on how patterns differ across readily observed subgroups of occupations, education levels, and gender. These analyses are typically motivated by assumed average usage differences, assumptions that are indeed validated in our data but that ignore within-group usage differences. On average, white-collar and tertiary-educated workers have substantially higher skill usage (see fig. S3).

**Fig. 4.**
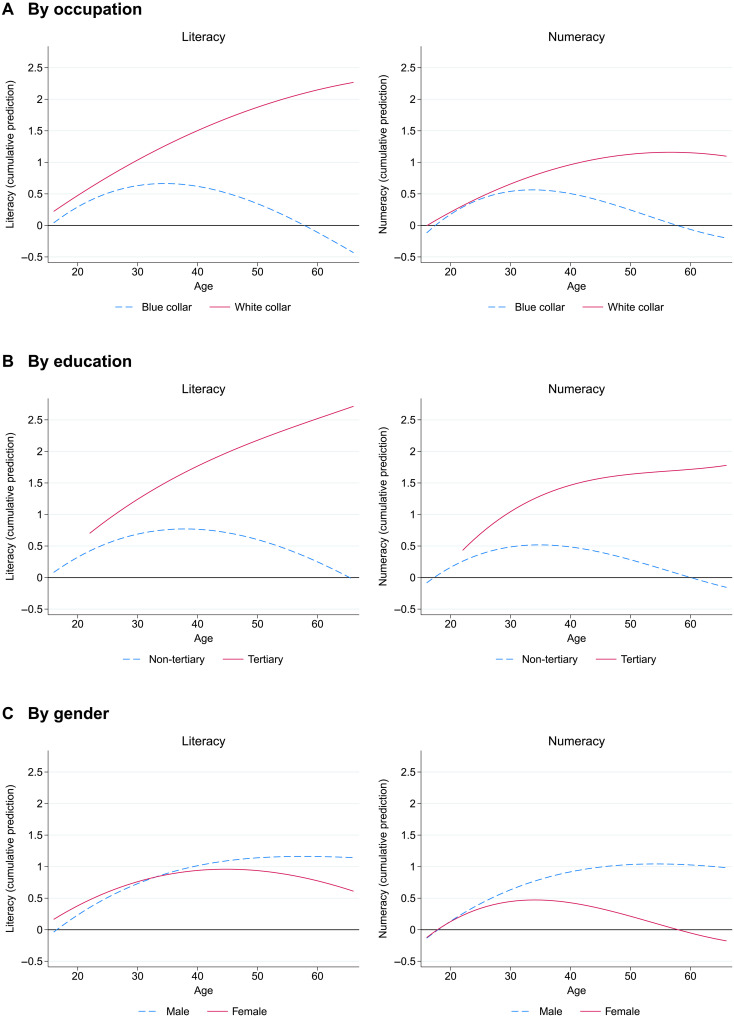
Age-skill profiles by background characteristics. Cumulative depiction of the predicted marginal annualized change in skills between the two waves at each age, adjusted for reversion to the mean. (**A**) By occupation. (**B**) By education. (**C**) By gender. Skills measured in SD units. Sample splits by blue-/white-collar occupations, (no) tertiary education, and gender, respectively. Sample: Employed workers, ages 16 to 65, weighted by sampling weights (*N* = 2497) (ages 22 to 65 for completed tertiary education). See figs. S10 to S12 for marginal skill changes by age. Data source: PIAAC-L.

These subgroup differences in usage show up in the age-skill patterns. Distinguishing between blue-collar and white-collar occupations yields results that are broadly similar to the overall split between low- and high-usage individuals. Skills consistently increase throughout the age range for white-collar workers but start to decline early for blue-collar workers (Fig. 4A). The increase for white-collar workers is more pronounced in literacy than in numeracy, particularly at older ages. Virtually the same pattern is observed when distinguishing between those with and without a tertiary education (Fig. 4B).

Interesting differences also appear by gender. In literacy, skill trajectories are initially similar across genders but then flatten out for men and decline slightly from the mid-forties for women (Fig. 4C). The numeracy pattern is similar for men, whereas for women, numeracy skills start to decline from their early thirties and do so more strongly. While the more pronounced gender differences in numeracy might be explained by the more frequent math usage of men (fig. S3), we show below that the differences go beyond that.

To summarize the aggregate difference in profiles by subgroup, we perform regression analyses that allow for multiple influences simultaneously (Table 1). The regressions express the change in skills as a quadratic function of age and approximate the subgroup effects by linear shifts in the skill changes for all ages. Going from no monthly usage of skills to at least monthly usage in all of the measured usage categories (i.e., from 0 to 1 on the usage index) is associated with an average annual increase in skills by a statistically highly significant 0.108 SD in literacy and 0.100 SD in numeracy (columns 1 and 6). The average difference in skill changes between blue- and white-collar workers is 0.056 SD in literacy and 0.030 SD in numeracy (columns 2 and 7). The age-skill pattern is shifted by 0.057 SD in literacy and 0.039 SD in numeracy between workers with and without tertiary education (columns 3 and 8) and by 0.014 SD in literacy and 0.026 SD in numeracy between men and women (columns 4 and 9). In general, coefficient estimates are reduced in size when the full set of measures is jointly considered, but they maintain their relative ordering and their significance (columns 5 and 10). The main exception is that the coefficient on white-collar occupations for numeracy becomes small and loses statistical significance.

**Table 1.** Heterogeneity in marginal changes in skills. Least squares regressions weighted by sampling weights. Dependent variable: Individual marginal annualized change in skills between the two waves, adjusted for reversion to the mean. Skills measured in SD units. Age squared divided by 1000. Skill usage: Average of indicators of at least monthly skill usage in different categories at work and at home. Sample: Employed workers, ages 16 to 65. Regressions use 10 plausible values of skill measurement per observation (individual). SEs clustered at the individual level in parentheses. Significance level: *** 1%, ** 5%, * 10%. Data source: PIAAC-L.

	Literacy					Numeracy				
	(1)	(2)	(3)	(4)	(5)	(6)	(7)	(8)	(9)	(10)
Age	−0.0041*** (0.0015)	−0.0047*** (0.0015)	−0.0052*** (0.0015)	−0.0033** (0.0015)	−0.0055*** (0.0015)	−0.0059*** (0.0018)	−0.0063*** (0.0019)	−0.0068*** (0.0018)	−0.0054*** (0.0018)	−0.0067*** (0.0018)
Age squared	0.030 (0.019)	0.034* (0.019)	0.039** (0.019)	0.020 (0.019)	0.043** (0.019)	0.054** (0.023)	0.055** (0.024)	0.060*** (0.023)	0.046** (0.023)	0.061*** (0.023)
Skill usage	0.108***(0.015)				0.055*** (0.016)	0.100*** (0.018)				0.074*** (0.018)
White-collar occupation		0.056*** (0.006)			0.034*** (0.007)		0.030*** (0.008)			0.011 (0.010)
Tertiary education			0.056*** (0.007)		0.030*** (0.008)			0.039*** (0.009)		0.024** (0.012)
Female				−0.014** (0.006)	−0.017*** (0.006)				−0.026*** (0.008)	−0.022*** (0.008)
Constant	0.065** (0.027)	0.115*** (0.027)	0.136*** (0.027)	0.119*** (0.028)	0.108*** (0.027)	0.115***	0.150*** (0.033)	0.165*** (0.033)	0.159*** (0.034)	0.142*** (0.033)
						(0.033)				
*R*^2^ (adj.)	0.046	0.050	0.048	0.020	0.070	0.036	0.025	0.029	0.023	0.046
Observations	2497	2497	2497	2497	2497	2497	2497	2497	2497	2497

Results are robust to using various alternative measures of skill usage in a regression framework (table S1). Skill usage at work and skill usage at home are significantly related to skill changes when entered individually (columns 1 and 2). When included jointly, both remain significant, but the coefficient estimates for usage at work are twice as large as for usage at home (column 3). When reading and math usage are included jointly, math usage is statistically insignificant in the literacy regression, but reading usage is significant in the numeracy regression (column 4). The skill-usage measures considered so far measure whether an activity is performed at least once a month. Alternatively, we can use the full information of the underlying five-point frequency scale from never to every day, linearizing the five options from zero to one. Coefficients on this alternative usage measure are even larger in both subjects (columns 5 and 6).

#### Post-40 skill trajectories by characteristics and usage

**Fig. 5.**
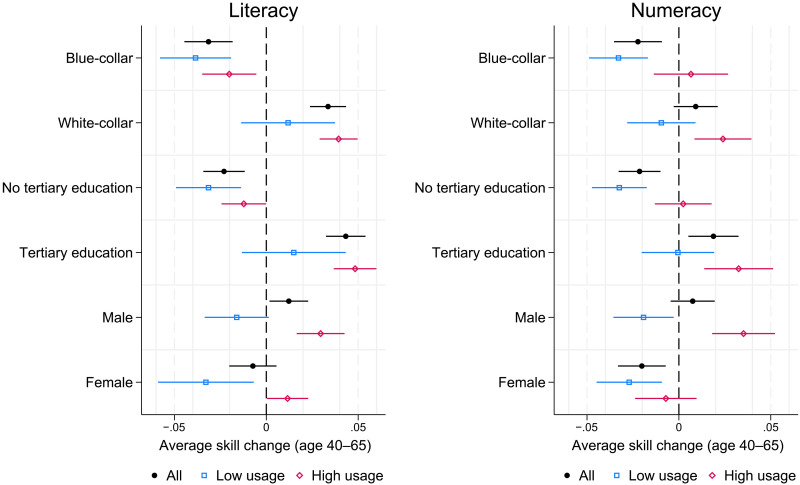
Skill changes after age 40: By background characteristics and skill usage. Average individual marginal annualized change in skills between the two waves, adjusted for reversion to the mean, and 95% confidence band. Skills measured in SD units. Positive values indicate increasing skills, negative values indicate decreasing skills. Subgroup means by blue-/white-collar occupations, (no) tertiary education, and gender, respectively. Low/high skill usage: Below/above median of skill usage at work and at home. Sample: Employed workers, ages 40 to 65, weighted by sampling weights. Data source: PIAAC-L.

Interpreting the age-skill patterns across the background factors is complicated by their interactions with varying amounts of skill usage (see fig. S3). To highlight these interactions, Fig. 5 plots the average change in skills in various subgroups for the population over age 40, roughly above the population mean age and where the previous analysis suggests that any differences should be apparent. Zero on the plot indicates no post-40 change in skills, recorded as average annual changes in SD units.

Within each of the subgroups, there are large differences in skill trajectories by whether individuals do or do not frequently use the respective skills. Even among those groups with average skill growth after age 40, white-collar and tertiary-educated workers, only individuals with frequent skill usage show increases. Individuals in these groups with below-average usage show no significant change. Similarly, in the groups previously showing average age-related declines, blue-collar and less-educated workers, there are no significant skill losses beyond age 40 for those with above average usage (except for blue-collar workers in literacy). In sum, skill gains or losses in the second half of working-age life are strongly mediated by the frequency of skill usage.

The aging pattern by gender is notably different. In literacy, usage dominates the overall age-skill relationship for both males and females. However, in numeracy, usage differences by females have noticeably smaller differential effects on age patterns. The age trajectory of numeracy skills for women is less dependent on usage, and only high usage arrests numeracy skill loss for women aged 40 to 65. This pattern is not driven by differences in the subgroup shares falling above or below the overall sample median of usage, as they emerge similarly when splitting the subgroups by the median in their respective subgroup (fig. S13).

These results are not dependent on the specific definition of the age sample. Qualitative results are similar in the smaller subsamples of workers aged over 45 or 50 for all subgroup categories.

## DISCUSSION

Cognitive skills of the population such as literacy and numeracy are important not only for individual incomes but also for the economic growth of nations (*2*–*6*). As a result, the aging of world populations presents an economic concern if the commonly assumed declines of these skills with age hold.

We use longitudinal variation in individual literacy and numeracy skills for a representative adult sample to create age-skill profiles that credibly separate age from cohort effects. The pure age component that we derive provides a different perspective on the impacts of aging populations. Overall, our results are not consistent with a view that a natural law dictates an inevitable decline in these skills with age. Potential cognitive declines only occur at later ages and are not inevitable with usage of skills.

This is consolation for countries with aging populations, but avoidance of skill losses is not automatic and appears related to stimulation from skill usage. These results thus suggest that age-skill relationships of adults deserve policy attention, consistent with concerns about the necessity of lifelong learning.

In the following, we discuss how our study relates to findings of alternative prior approaches. We then consider the limitations of our work.

### Separating age from cohort effects in representative samples

A series of studies of age-skill patterns has come from prior international adult surveys covering more limited sets of countries: the 1994–1998 International Adult Literacy Survey (IALS) and the 2003 Adult Literacy and Life Skills Study (ALL). While these studies consider skills of nationally representative samples, they rely on purely cross-sectional information for estimating age-skill patterns. Thus, they require the strong assumption that individuals born and educated over a long period of time are otherwise similar in the factors affecting cognitive skills [for details of the various studies, see the review by (*1*)]. Because this approach relies on observed group differences in skills by individuals of different ages, it is naturally impossible to disentangle individual aging patterns from underlying cohort differences.

The empirical differences in conclusions are striking. Cross-sectional patterns show decreasing average skills starting early in adulthood, but our age-skill patterns based on longitudinal data lead to the conclusion that average skills increase through middle age.

By combining IALS, ALL, and PIAAC data, it is possible to create synthetic cohorts that allow tracing skills over time for separate representative samples of birth cohorts (*10*, *18*, *19*). While conceptually superior to the pure cross-sectional estimates, a variety of issues including changes in the underlying sampled populations, varying employment patterns, and comparisons of disparate tests that are not psychometrically linked complicate any analysis of age-skill patterns, leading to possibly erroneous conclusions from synthetic cohort methods (*20*). By contrast, our analysis is able to track the actual cognitive skill changes of individuals that are seen with aging on the same psychometric testing instrument.

Without our focus on economically relevant skills, many studies on cognitive aging have used longitudinal data for individuals, but they also tend to come from nonrepresentative samples. For example, the groundbreaking Seattle Longitudinal Study follows convenience samples from a Health Maintenance Organization in Washington State since 1956 (*15*). This development of rich panel data on individuals permits a range of detailed psychological investigations. However, limited representation may constrain generalizability from skill changes observed in the selected sample, not least because our results show that age-skill patterns differ markedly with behavioral and contextual factors. The focus of investigation on various components of intelligence and other measures of development also prevents ready translation to the economic skills that motivate our work.

In the context of international testing, an exception to the cross-sectional data is a small second testing of the Swedish IALS data that uses a slightly different follow-on assessment and provides 622 usable retests in 1998 for the original 3038 observations in 1994. This has been used to study skill depreciation during unemployment (*21*), but it is difficult to generalize to overall age-skill profiles.

### Theories of cognitive aging

The extensive body of psychological and neuroscience research into cognitive aging indicates that age patterns can differ markedly for different components of cognitive functioning (*1*, *11*, *14*). This research provides clues to the underlying causes of the patterns that we document in economically relevant skills. Although much of the interpretation of the interplay between social/behavioral aspects and physical components of aging is currently in flux, this work tends to be consistent with the patterns we observe.

While findings vary, both cross-sectional and longitudinal studies tend to show differing patterns for components related to fluid intelligence (independent of prior learning) and components related to crystallized intelligence (deduced from prior learning) (*13*, *15*, *22*, *23*). In broad terms, this literature finds that cognitive components related to fluid intelligence, such as attentional and memory capacity, processing speed, and spatial orientation, start to decline in early adulthood (*16*, *24*, *25*). These changes imply that at older ages, it is more challenging to switch between tasks or solve complex numerical problems, which may affect numeracy skills in particular. Components related to crystallized intelligence, such as vocabulary knowledge and the processing of general information, tend to peak at later ages, often increasing until age 50 and only stagnating afterward (*25*). Our results suggest that the age trajectories of literacy and numeracy skills combine aspects of the age trajectories shown for components of fluid and crystallized intelligence. While these distinctions are not unambiguous, differences in relative weightings of these components may be showing up in the domain-specific age-skill patterns that we find between literacy and numeracy (Fig. 2). As discussed below, however, the precise relationship between the fundamental components of intelligence and our economically relevant skill measures is unclear.

The rapidly evolving neuroscience research on cognitive aging indicates that part of the age-skill patterns may be related to neurological alterations in aging brains (*12*, *26*, *27*). Research suggests that the volume of gray and white brain matter tends to fall (*28*), and this varies by brain region (*24*, *29*). In addition, age-related declines in frontal lobe function, particularly in regions related to inhibitory control, may reduce the ability to suppress distractions and irrelevant information with advancing age (*30*, *31*). At this point, it is difficult to assess how much of the age-skill patterns that we observe are related to neurocognitive aging.

### The role of context factors

A central finding of our analysis is that age-skill profiles differ significantly by skill usage. This finding is closely related to psychological and neuroscience research showing that cognitive aging is not inevitable but instead depends on social and cultural context factors as well as individuals’ behavior and genetics (*1*, *16*, *32*). Most closely connected to our analysis, exposure to or lack of challenging tasks at work have been shown to be associated with structural brain neuroplasticity, neurocognitive impairment, and performance in memory-based tasks at older age (*33*, *34*). In low-complexity jobs, repeated exposure to novel work tasks has been found to be related to higher levels of processing speed, working memory, and greater gray matter volume in various brain regions up to 17 years later (*24*). These findings are consistent with our result that heterogeneity in the population based on individual behavior, in particular skill usage, is the most notable aspect of age-skill patterns in literacy and numeracy.

In contrast to male numeracy skills, female numeracy skills fall with age, a difference that cannot be entirely explained by math usage. Some neuroscience research suggests these patterns may be related to gender differences in genetic and biosocial factors and their interactions with behavioral factors in older age (*11*). Studies have shown some gender differences in brain structure, chemistry, and function; on average, men tend to have larger brain volumes, while women tend to have relatively more gray matter and a higher cerebral blood flow, in addition to other neurochemical differences (*35*–*37*). Regarding brain aging, gender differences have been found in volume loss for specific brain regions, and gray matter volume seems to decline more strongly for men than for women (*35*). These patterns are also studied in relation to gender differences in risk for brain disorders such as women’s larger risk of Alzheimer’s disease (*38*). While difficult to link directly to our economically relevant skill measures, these studies suggest that there may be a neurological foundation for some of the gender differences that we find.

Gender differences in the level of performance on various cognitive components have been extensively studied. Men on average tend to have better mathematical reasoning and higher spatial ability than women, with gaps widening with age (*39*–*41*). Women on average tend to perform better in reading comprehension and writing (*42*). Some studies suggest that these differences have narrowed over time (*41*, *43*), while others find no significant change of gender gaps in large-scale representative samples (*42*). Linkage of these differences to aging, as well as generalizations to the longitudinal age structure of literacy and numeracy patterns, are less clear.

### Limitations

The restriction of our data to the age range 16 to 65 is an important limitation of our analysis. A similar analysis of the trajectory of literacy and numeracy skills in a representative sample of the population aged over 65 therefore remains an important topic for future research, albeit one less related to economic outcomes because of the much lower labor-force participation of this group. The difference in skill changes by usage in the age range studied here may offer important hypotheses for skill changes at older ages. Similarly, the usage aspect may be related to the resilience against age-related decline observed among cognitive super-agers who are able to maintain exceptionally high skills at older ages (*44*, *45*).

Another limitation is that our analysis is implemented only for Germany. It is an open question to what extent the patterns generalize to other countries. The cross-sectional age-skill patterns are quite similar between Germany and the pooled OECD sample (Fig. 1 and fig. S1), which may suggest comparability. Still, the fact that age-skill trajectories differ by individual backgrounds and behavioral contexts suggests that specifics of age-skill profiles differ between cultural, social, and environmental contexts. Generalizability to other populations therefore is an important direction for future research.

Our analysis focuses on literacy and numeracy as two important concepts of cognitive skills. These are skills that schools have identified as critical to students’ cognitive development, and they form the basis in many countries for testing and accountability of individuals and schools (*46*). In developing the PIAAC test, the OECD has independently focused on these concepts from analyses of labor-market demands and societal participation (*47*). It bases the testing decisions on readily measurable skills that have been validated in analyses of economic outcomes (*3*). While there is no sense that literacy and numeracy skills incorporate all of the skill dimensions that may be important in employment, there is strong evidence that they capture critical dimensions of employability and social integration.

While limited, there has been some attempt to consider how the cognitive skill measures of literacy and numeracy relate to fundamental psychological formulations of fluid and crystallized intelligence. This work suggests that our tested measures combine the underlying skill dimensions in complicated ways. Prior research on employment skills such as communication, mathematics, problem solving, and interpersonal skills, as well as psychological theories of crystallized and fluid intelligence, entered into the underlying theoretical development of the ALL test on which the conceptual framework of PIAAC is built (*47*–*50*). No attempt was made, however, to develop a precise crosswalk of these concepts. Therefore, it is not clear a priori whether literacy and numeracy skills should be expected to decline with age early on as with fluid intelligence or increase until later ages as with crystallized intelligence.

Our development of the full age-skill profile of literacy and numeracy skills relies on concatenating the marginal age changes across individuals of differing age. As such, it is not immune to possible cohort biases since with the sampling of individuals at a given time, respondents of different ages attended school and developed their cognitive skills at different times. The approach implicitly assumes that the development and implications of growing up at different times affect the level of skills but not the rate of change with age over the lifecycle. In other words, if cohort factors such as changes in school quality or motivation toward schooling change the scores for a cohort by a constant amount, the approach still yields unbiased estimates of the age-skill profile.

Testing this assumption is very difficult and little prior work is informative on it. Conceptually, we think of two major ways that age-specific contexts could affect the rate of skill change. First, at any point in time, individuals are observed in different situations. A leading example of age-varying situations that can be analyzed within our data is differences in employment. A major part of the social environment of workers is the workplace environment, and cognitive stimulation at work (going beyond the usage measures we have) may enter into the age-skill pattern. Retirement has been shown to affect skill trajectories (*51*–*53*). However, within our data, the results on aging and usage are very similar for the employed (Fig. 3) and the full sample (fig. S8). This suggests that our main pattern of results is not driven by labor-market contexts, at least in the age range observed in our analysis. Of course, there are many other context differences that could have systematic effects across our panel observations. While beyond possible analysis with our available data, expanded panel data drawing samples from different social and economic contexts would provide for testing of differing age-skill profiles.

Second, historical situations such as school structure and curriculum may differ for different cohorts, and this could in principle affect not just later skill levels but also their changes at different ages. This impact of historical factors is both more speculative and more difficult to test. For example, if there are changes over time in the extent to which school curricula convey the ability for lifelong learning, the apparent age-skill pattern may partially reflect contextual differences. The difficulty is that our data do not allow us to separate age from these contextual factors because we only observe skill changes of differently aged individuals at one point in time. The impact of historical context could potentially be tested by observing same-aged individuals with known different historical contexts at one point in time or alternatively by observing the skill change of different cohorts when they are the same age. Future surveys that incorporate these characteristics could provide for the testing of this basic assumption.

A limitation of the analysis of how age-skill patterns differ by skill usage is that the descriptive analyses of observational data do not directly allow for a causal interpretation of the estimated associations. Relating differences in the usage of skills and other characteristics observed at the first testing occasion to subsequent changes in skills shields estimates from outright reverse causation. However, the behaviors and characteristics are not distributed randomly, i.e., they may be associated with unobserved omitted factors that are independently related to skill trajectories.

One place where the interplay of usage and other observable behavioral differences is particularly observable involves physical/health behaviors and outcomes. Physical activity (*54*, *55*), social activity (*56*), healthy and nutritious diets (*57*, *58*), and better health status (*59*) have been identified in prior literature as being related to slower cognitive decline. Consistent with this evidence, when distinguishing the age-skill profiles of literacy and numeracy by available measures of lifestyle modulations, cognitive aging patterns differ in ways that resemble the split between low- and high-usage individuals. In particular, literacy and numeracy skills tend to increase into older ages for individuals with high, but not low, sports activity and health status, with divergent patterns being more pronounced for literacy than numeracy (figs. S14 and S15). Further, these average patterns parallel the average usage of each skill across the same categories (fig. S16). However, cognitive skill trajectories after age 40 still differ strongly by skill usage within subgroups of sports activity and health status, reinforcing our emphasis on skill usage (fig. S17). These patterns are further seen in data on alcohol consumption. Individuals with relatively high alcohol consumption show more positive age trajectories of literacy and numeracy skills beyond the early forties than individuals with relatively low alcohol consumption (fig. S18). This pattern matches the relatively higher usage of both skills by high alcohol consumers (fig. S16). Skill trajectories with age, however, again differ by usage within subgroups of alcohol consumption (fig. S17). When considering these factors jointly with skill usage in a regression framework similar to Table 1, the impact of skill usage remains large and significant (table S2). Only sports activity (but not health status or alcohol consumption) enters significantly, and its impact is much smaller than skill usage. Thus, we conclude that the effect of usage is not strongly moderated by these other factors. These descriptive patterns do not have causal interpretation, but their consistency is notable, and they open directions for future research into underlying mechanisms.

## MATERIALS AND METHODS

### PIAAC data

Our analysis relies on the unparalleled collection of longitudinal skill data for a representative adult population. The underlying PIAAC assessments include measures of skills that are related to life and work outcomes; the samples cover the full adult population; and the data provide information about the background and employment of individuals.

#### PIAAC-Longitudinal

Two waves of repeated testing of the same individuals took place in Germany in 2011/2012 and 2015. The first wave was part of PIAAC, an international cross-sectional adult test of literacy and numeracy skills administered by the OECD in 39 countries (*8*, *60*). PIAAC samples are drawn to be representative of the population aged 16 to 65 in each country.

In Germany, the PIAAC-Longitudinal (PIAAC-L) project returned to the original participants to create a panel study (*61*). Canada, Italy, and Poland also implemented follow-on surveys of the original PIAAC participants, but they did not repeat the skill test (*61*). PIAAC invested substantial effort in skill measurement, with an average testing time of roughly 60 min and an additional 40 min for answering a background questionnaire (*62*). Operationally, data collection was performed under the supervision of trained interviewers in respondents’ homes using a computer-based application (with the option of using a paper version) that started with the background questionnaire and followed with the skill test.

The PIAAC assessment is designed to test skills that are relevant for adults to participate in social life and work situations, as opposed to specific components of cognitive functioning or purely curriculum-based tests. Skills are tested in two main domains, literacy and numeracy. PIAAC defines literacy as the “ability to understand, evaluate, use, and engage with written texts in order to participate in society, achieve one’s goals, and develop one’s knowledge and potential” (*60*). Numeracy is defined as the “ability to access, use, interpret, and communicate mathematical information and ideas in order to engage in and manage the mathematical demands of a range of situations in adult life.” The original PIAAC assessment also included the optional (for countries and individuals) domain of “problem solving in technology-rich environments” for the subsample of participants with confident computer usage, but this domain was not included in PIAAC-L.

PIAAC-L administered the literacy and numeracy tests under conditions identical to the international PIAAC setting. The average time between the first and second test is 3.56 years. From the 5379 initial participants from 2012, a sample of 3263 participants (60.7%) was tested again in 2015 (*61*, *63*). An analysis of the original and retaker samples does not indicate that selection issues from decisions to participate in the second survey are likely to bias our age-skill estimates. Comparing the cross-sectional age-skill profiles for the two samples using the original sampling weights (fig. S19A), retakers are slightly positively selected in terms of achievement, a phenomenon generally consistent with other longitudinal assessment surveys (*64*). Reassuringly for our purposes, however, this positive selection does not differ by age, with observed age-skill profiles shifted upward in parallel. The differences disappear when using the PIAAC-L sampling weights to adjust the retaker sample to the general population in terms of observables (fig. S19B). Thus, the sampling weights (used throughout our analysis) ensure depictions that are representative of the full population.

To allow for joint analysis of achievement on the two test occasions, PIAAC-L uses Item Response Theory (IRT) scaling procedures to provide 10 plausible values of achievement measures for both waves. We use all 10 plausible values throughout our analysis, clustering SEs at the level of individuals. For analytical purposes, we standardize scores to have mean zero and SD one in the 2012 test for the retaker sample.

The average age in our analysis sample is 41.3 years (table S3). A total 37.8% have a white-collar occupation [measured by codes 1 to 4 of the International Standard Classification of Occupations (ISCO): managers, professionals, technicians and associate professionals, and clerical support workers]. A total of 30.6% have tertiary education. The intertemporal test data indicates that literacy scores are on average 0.049 SD higher and numeracy scores are 0.020 SD higher on the second test compared to the first test. These positive average changes, observed within individuals, already cast doubt that the overall negative slopes of the cross-sectional age-skill profiles in Fig. 1 reflect actual age effects. It is very unlikely that observed skill changes reflect learning from past test responses (*65*): (i) because of the long time (3 to 4 years) between the two tests, (ii) because most participants did not get identical test items given the rotation of test booklets that underlies the IRT scaling, and (iii) because participants were neither informed about their first scores nor received feedback on their answers.

Most of our analysis focuses on the employed (2497 observations) for whom we can observe skill usage patterns both at work and at home. For this sample, average achievement as well as shares of tertiary education and white-collar occupations are somewhat higher. Nonetheless, the overall age-skill patterns we analyze follow very similar patterns as those in the full sample (fig. S4). The cross-sectional age-skill pattern in the employed sample indicates a decline in literacy starting in the early thirties and in numeracy starting in the early forties (fig. S20).

#### Skill usage

In addition to measuring skills, the initial PIAAC wave collects rich information on individuals’ usage of skills. The background questionnaire includes separate item batteries on the frequency of respondents’ activities related to reading and to math at work and in everyday life. The reading usage items refer to the frequency of reading various types of documents, and the math usage items refer to the frequency of various activities involving numbers and math. In both domains, the usage measures that we construct contain six items, and the same items are used for skill usage on the job and in everyday life. The six reading items refer to how often respondents usually (1) read directions or instructions; (2) read letters, memos, or e-mails; (3) read articles in newspapers, magazines, or newsletters; (4) read articles in professional journals or scholarly publications; (5) read books; and (6) read manuals or reference materials. The six math items refer to how often respondents usually (1) calculate prices, costs, or budgets; (2) use or calculate fractions, decimals, or percentages; (3) use a calculator; (4) prepare charts, graphs, or tables; (5) use simple algebra or formulas; and (6) use more advanced math or statistics (calculus, trigonometry, and regressions). There are two additional items in the PIAAC reading usage battery that might reflect math usage: read bills, invoices, bank statements, or other financial statements and read diagrams, maps, or schematics. We do not include these items in our literacy usage measure, but qualitative results are identical if we do. In addition, there is a separate battery of four items on writing activities. If we combine these items with the reading items, qualitative results are again the same.

For each item, the calendar-based usage frequency is measured on a five-point scale from never to every day. We collapse the separate categories into a composite measure by detecting whether individuals perform each given activity at least once a month and then taking a simple average over the six work items and the six home items of reading and math usage, respectively. Results are similar when linearizing across the five frequency-of-use categories rather than focusing on at least monthly usage (table S1, columns 5 and 6).

The usage of skills varies widely by domain and background characteristics. On average, respondents perform 59.7% of the reading activities and 35.3% of the math activities at least once per month. Workers in white-collar occupations use skills much more frequently than workers in blue-collar occupations (69.7% versus 49.7% in reading and 43.2% versus 27.3% in math); see fig. S3 for details. Similar differences exist between respondents with and without tertiary education. While there is no gender difference in reading usage, men perform math activities more frequently than women (38.2% versus 32.0%). Most of these usage patterns do not differ strongly between workers above or below the sample median of age (fig. S3). The main exception is that the math (and to a lesser extent reading) usage of blue-collar workers and of individuals without tertiary education declines with age. Overall reading usage is more frequent at home than at work (65.2% versus 54.2%), whereas the opposite is true for math usage (32.8% versus 37.7%). The correlation between skill usage at work and at home is 0.411 for reading and 0.343 for math. Reading and math usage are correlated at 0.506. Skill usage is also significantly correlated with test scores (0.357 for reading usage and literacy and 0.401 for math usage and numeracy).

### Empirical approach

#### Changes in skills in individual longitudinal data

Our analysis uses individual data about how literacy and numeracy scores change over a three-to-four-year period to trace how skills change within individuals along the age spectrum. We observe measures of skills, *T_ia_*, for individual *i* at age *a* along with a second measure of the same skills at age a*. We are interested in estimating the average change in skills as individuals move from *a* to a*. In the absence of measurement error, one could take the simple average of the individual differences in skills at any given age

$${∆}_{a}=T_{ia^{*}}-T_{\mathrm{ia}}$$ (1)

These skill changes, observed within individuals, are not confounded with cohort effects.

#### Adjustment for measurement error due to reversion to the mean

Analyzing the pattern of changes in skills for individuals, however, leads to immediate measurement issues. The complication is that the test-score measure of skill comes with error such that

$$T_{\mathrm{ia}}={\tilde{T}}_{\mathrm{ia}}+\varepsilon_{\mathrm{ia}}$$ (2)

where ${\tilde{T}}_{\mathrm{ia}}$ is the true cognitive skill of the person and $\varepsilon_{\mathrm{ia}}$ is the test measurement error. Similarly, we also only observe T_ia*_ measured with error.

The presence of measurement error complicates the estimation of age effects even when the errors have mean zero and constant variance. This problem was addressed as early as the inheritance studies of Galton (*66*) and has been formalized in (*9*). We define the true difference in cognitive skills as

$${\tilde{∆}}_{a}=E\left({\tilde{T}}_{ia^{*}}-{\tilde{T}}_{\mathrm{ia}}\right)$$ (3)

Assuming that the observed *T_ia_* and T_ia*_ are distributed bivariate normal with a mean of μ for *T_ia_* and a correlation of ρ, (*9*) derive that

$$E\left(T_{ia^{*}}-T_{\mathrm{ia}}|T_{\mathrm{ia}}\right)={\tilde{∆}}_{a}-\left(1-\rho \right)\left(T_{\mathrm{ia}}-\mu \right)$$ (4)

This relationship shows that for observed cognitive skills below the mean (i.e., $T_{\mathrm{ia}}<\mu$), the true difference in skills will tend to be overestimated, while the opposite is the case for observations above the mean.

This overall pattern makes intuitive sense. If there is no measurement error (i.e., ρ = 1), the difference in observed values equals the true marginal age effect, ${\tilde{∆}}_{a}$. If, however, there is measurement error, observations far below the mean are likely to be observations that have larger negative measurement errors. These errors are unlikely to be duplicated with similarly large negative errors at the second measurement (a*), meaning that the farther an observation is below the mean, the larger the positive bias in estimated differences. The opposite holds for observations above the mean, for which the estimated differences will be negatively biased. If uncorrected, this phenomenon would bias the observed age-skill patterns whenever true skills differ by age. In practice, estimating the age-skill patterns with unadjusted data yields distorted pictures (fig. S9).

The assessment of this bias, referred to as reversion to the mean (also called regression to the mean or simply regression effect in various analyses), yields a natural way to estimate the error-corrected change in cognitive skills from our observed data

$${\hat{\Delta }}_{\mathrm{ia}}=\left(T_{{\mathrm{ia}}^{*}}-T_{\mathrm{ia}}\right)+\left(1-r\right)\left(T_{\mathrm{ia}}-{\bar{T}}_{a}\right)$$ (5)

where *r* and ${\bar{T}}_{a}$ are the sample analogs of ρ and μ. This adjustment is used throughout our analysis of patterns in cognitive score changes.

Equivalently, the same adjustment can be obtained by taking the residuals from a regression of the test-score change on the initial test-score level. We also experimented with an alternative adjustment that conditions on a full set of age fixed effects in the regression of skill change on initial skill level. The idea is to retain any variation in skill changes that is related to age while taking out the part of the variation related to the initial skill level that is not related to age. Results using this alternative method are nearly identical.

#### Age-skill patterns with adjusted data

Our goals are to use the individual data to estimate the age-skill pattern and to study how this pattern relates to individual characteristics, behavior, and circumstances. The basic approach is to cumulate the adjusted marginal skill changes (${\hat{\Delta }}_{\mathrm{ia}}$) across the age distribution and to estimate the impact of key individual differences including skill usage and other circumstances across the life cycle.

We can estimate the aggregate age-skill pattern by adding the cumulative averages of (annualized) adjusted marginal changes in skills at each age to the starting skill level at age 16

$${\hat{T}}_{a}={\bar{T}}_{i16}+\sum_{\alpha =16}^{a}{\bar{\hat{\Delta }}}_{\alpha }$$ (6)

where ${\hat{T}}_{a}$ is the adjusted aggregate skill level for individuals with age = *a* and ${\bar{\hat{\Delta }}}_{\alpha }$ is the average marginal (1-year) change across all individuals with age equal to *a*. Plotting these adjusted scores allows us to produce Fig. 2, which is an adjusted version of Fig. 1 based on longitudinal data. When we compare subgroups in the depictions of Figs. 3 and 4, we account for initial differences in skill levels by predicting scores at age 16 from a regression of scores on a quadratic age term within the respective subgroup in the initial wave.

The subsequent analysis of how age-skill patterns vary with individual backgrounds, behavior, and characteristics can be depicted by relating these factors to the adjusted individual skill data. A stylized version of our basic analytical specification is

$${\hat{\Delta }}_{\mathrm{ia}}=\gamma_{0}+\gamma_{1}a+\gamma_{2}a^{2}+f\left(U_{\mathrm{ia}}\right)+g\left(X_{\mathrm{ia}}\right)+\nu_{\mathrm{ia}}$$ (7)

where the γs are parameters of a quadratic age impact on cognitive skills, $f\left(U_{\mathrm{ia}}\right)$ considers usage of skills and $g\left(X_{\mathrm{ia}}\right)$ other systematic influences, and ν adds a stochastic term. The regressions in Table 1 follow this specification.
