# Supplementary material for: Age and cognitive skills: Use it or lose it
Source: Sci Adv. 2025 Mar 5;11(10):eads1560. doi: 10.1126/sciadv.ads1560 (PMC11881919; doi:10.1126/sciadv.ads1560)
Supplement: Supplementary file 1 — Figs. S1 to S20 Tables S1 to S3 [file sciadv.ads1560_sm.pdf]

Science Advances
AAAS

Supplementary Materials for

Age and cognitive skills: Use it or lose it

Eric A. Hanushek *et al.*

Corresponding author: Eric A. Hanushek, hanushek@stanford.edu

Sci. Adv. **11**, eads1560 (2025)
DOI: 10.1126/sciadv.ads1560

This PDF file includes:

Figs. S1 to S20
Tables S1 to S3

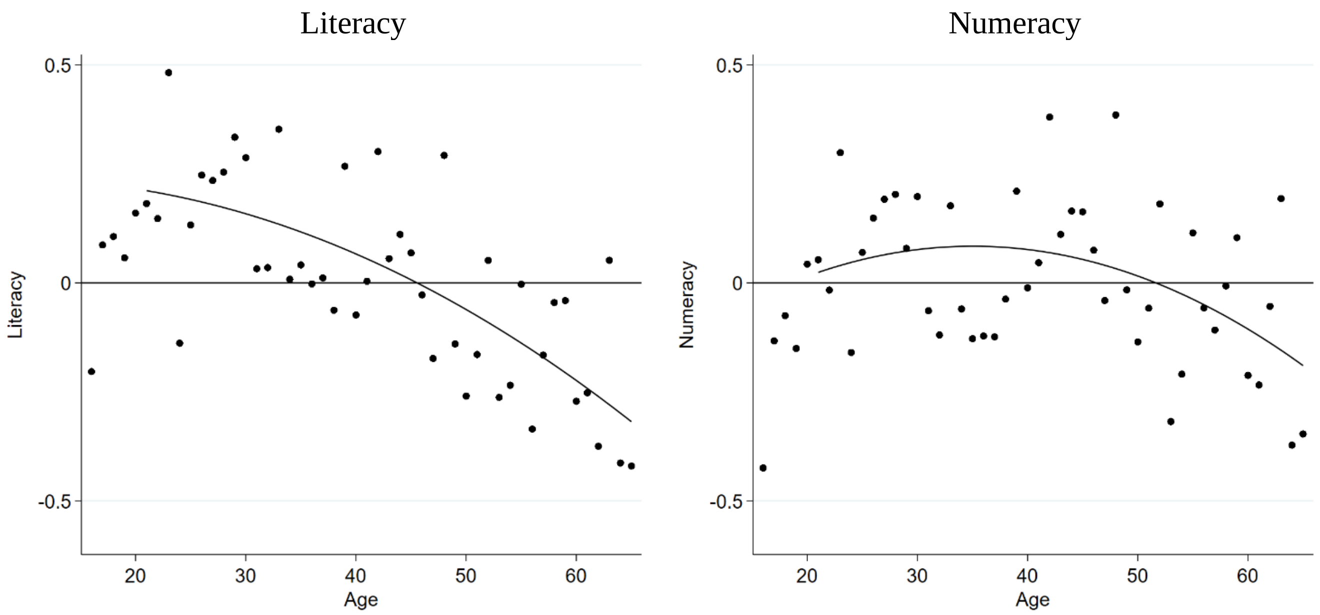

Fig. S1. Cross-sectional age-skill profiles: German PIAAC-L sample. Cross-sectional association between age and skills in the initial (2012) wave. Dots: average skills by age. Line: quadratic fit (estimated over 21-65 age range). Skills measured in SD units. Sample: full population, ages 16-65, weighted by sampling weights (N = 3,263). Data source: PIAAC-L.

A Cumulative age-skill profiles

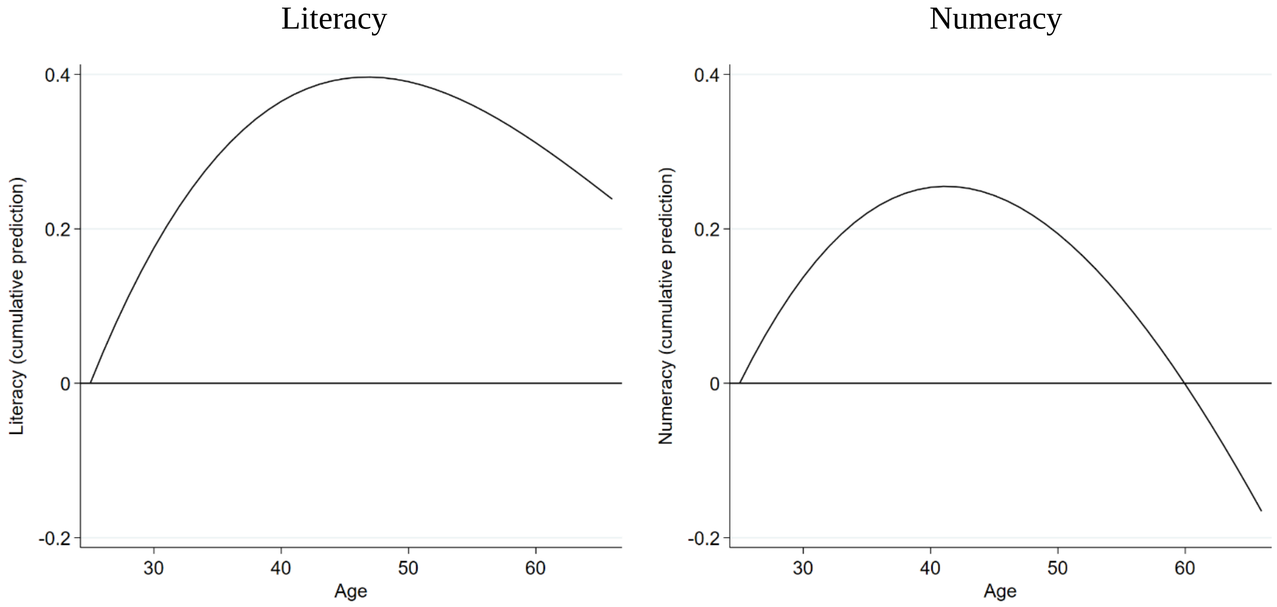

B Marginal skill changes by age

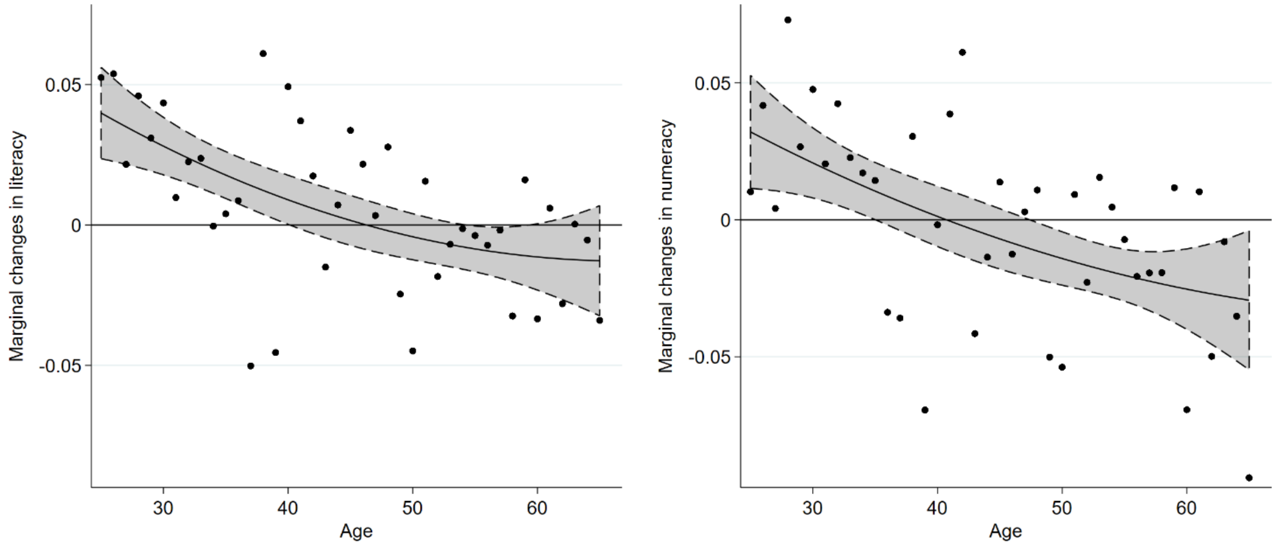

Fig. S2. Longitudinal age-skill profiles: Sample aged 25-65. Panel A: cumulative depiction of the predicted marginal change in skills at each age. Panel B: marginal annualized change in skills between the two waves by age, adjusted for reversion to the mean. Dots: average individual marginal annualized change in skills by age. Line: quadratic fit. Gray area: 95 percent confidence interval. Skills measured in SD units. Sample: full population, ages 25-65, weighted by sampling weights (N = 2,664). Data source: PIAAC-L.

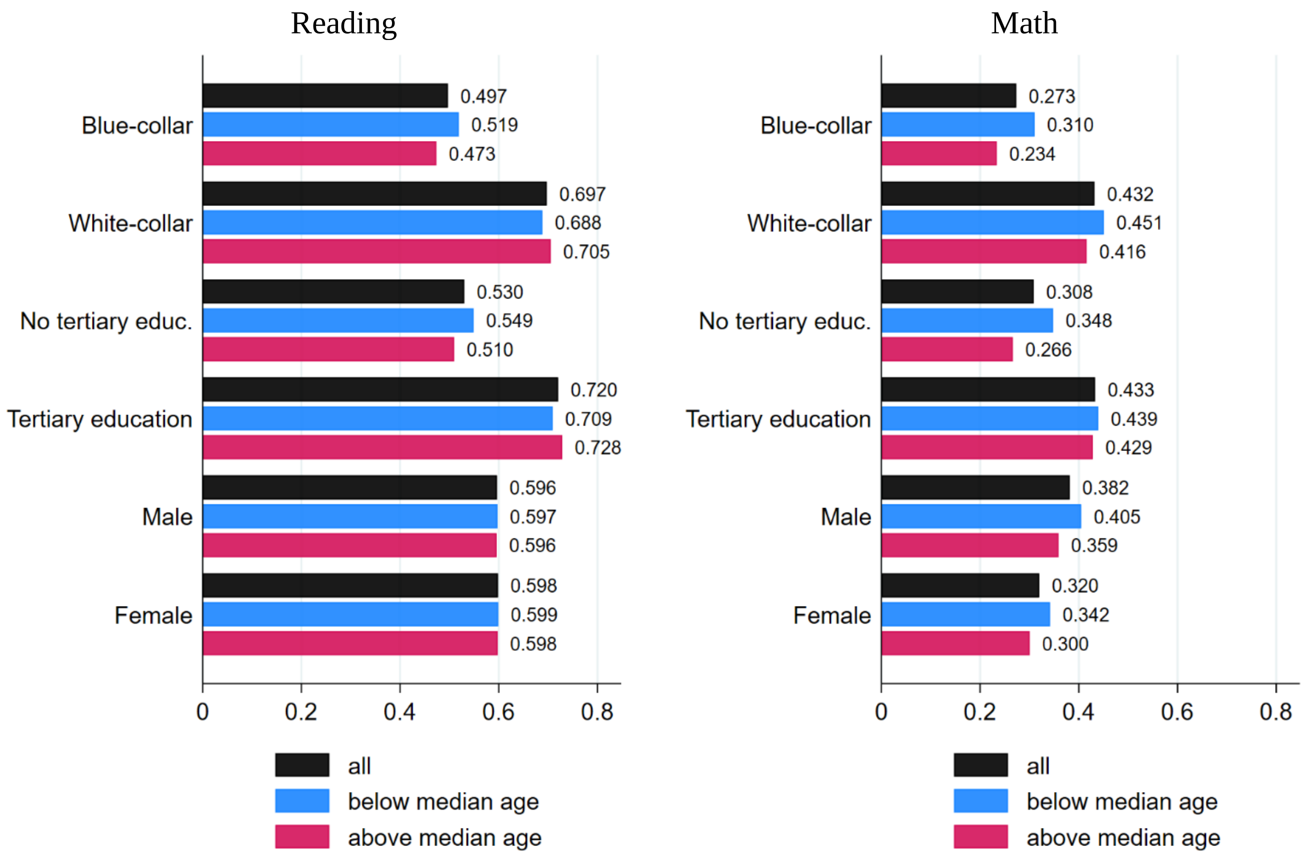

Fig. S3. Skill usage: By background characteristics and age. Average of indicators of at least monthly skill usage in different categories at work and at home. Subgroup means by blue-/white-collar occupations, (no) tertiary education, and gender, respectively. Below/above median age: sample split by median of age (43). Sample: employed workers, ages 16-65, weighted by sampling weights. Data source: PIAAC-L.

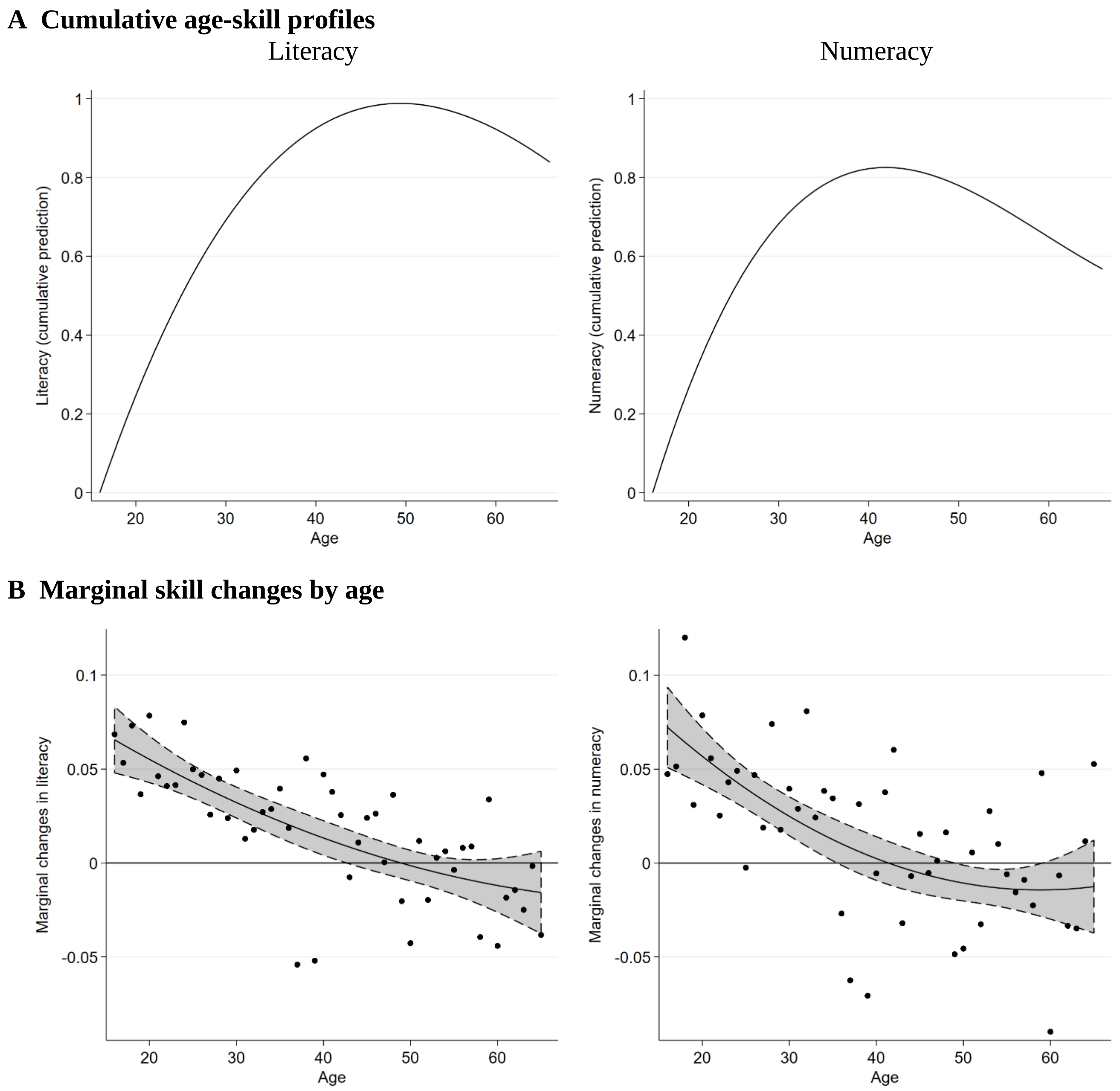

Fig. S4. Longitudinal age-skill profiles: Employed sample. Panel A: cumulative depiction of the predicted marginal change in skills at each age. Panel B: marginal annualized change in skills between the two waves by age, adjusted for reversion to the mean. Dots: average individual marginal change in skills by age. Line: quadratic fit. Gray area: 95 percent confidence interval. Skills measured in SD units. Sample: employed workers, ages 16-65, weighted by sampling weights (N = 2,497). Data source: PIAAC-L.

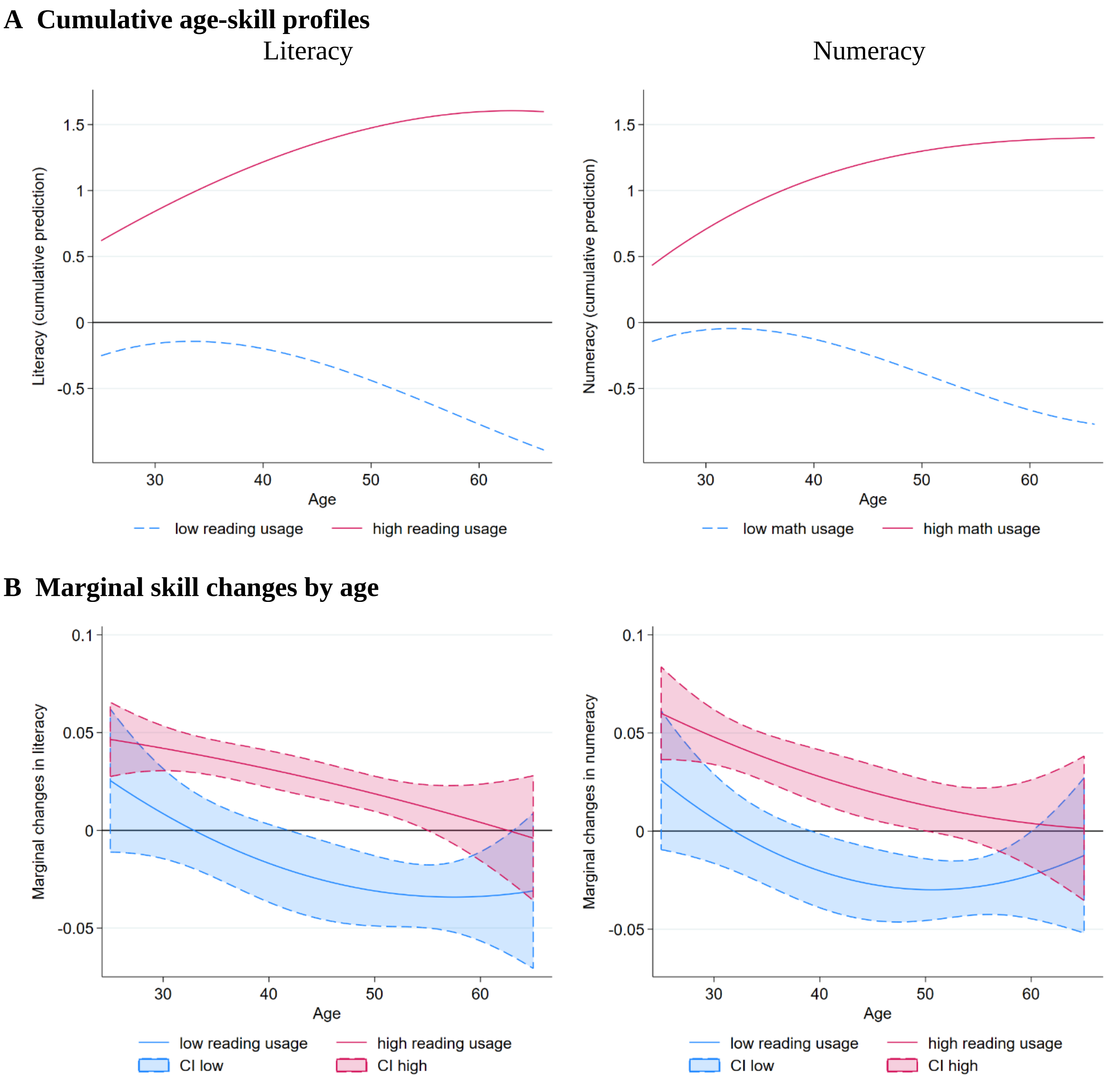

Fig. S5. Age-skill profiles by skill usage: Sample aged 25-65. Panel A: cumulative depiction of the predicted marginal change in skills at each age. Panel B: quadratic fit (with 95 percent confidence interval) of marginal annualized change in skills between the two waves by age, adjusted for reversion to the mean. Skills measured in SD units. Sample split by median of skill usage at work and at home. Sample: employed workers, ages 25-65, weighted by sampling weights (N = 2,178). Data source: PIAAC-L.

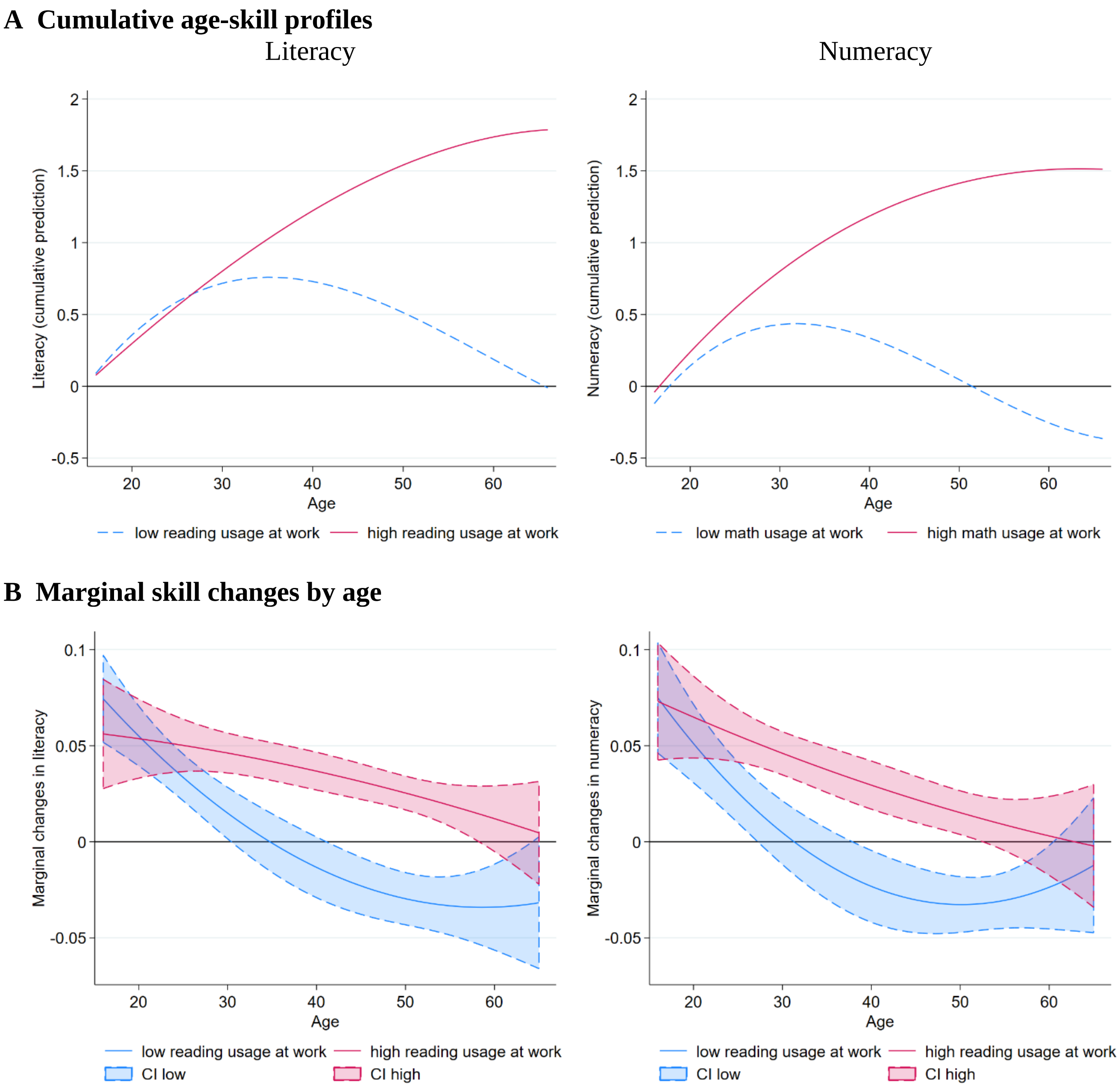

Fig. S6. Age-skill profiles by skill usage at work. Panel A: cumulative depiction of the predicted marginal change in skills at each age. Panel B: quadratic fit (with 95 percent confidence interval) of marginal annualized change in skills between the two waves by age, adjusted for reversion to the mean. Skills measured in SD units. Sample split by median of skill usage at work. Sample: employed workers, ages 16-65, weighted by sampling weights (N = 2,497). Data source: PIAAC-L.

A Cumulative age-skill profiles

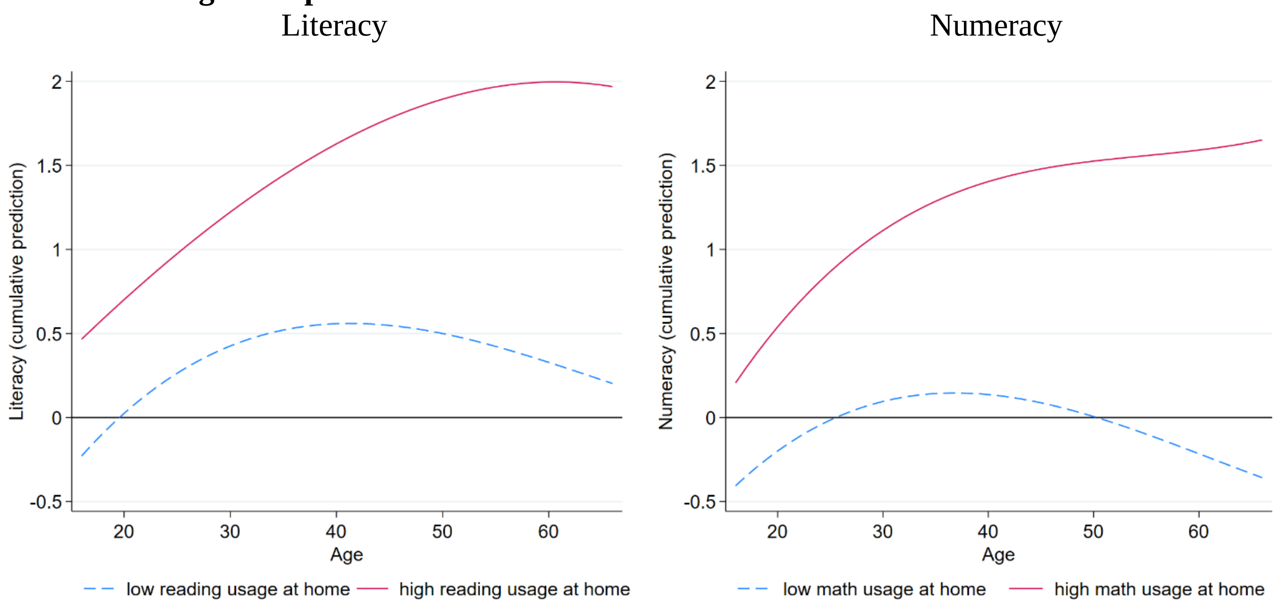

B Marginal skill changes by age

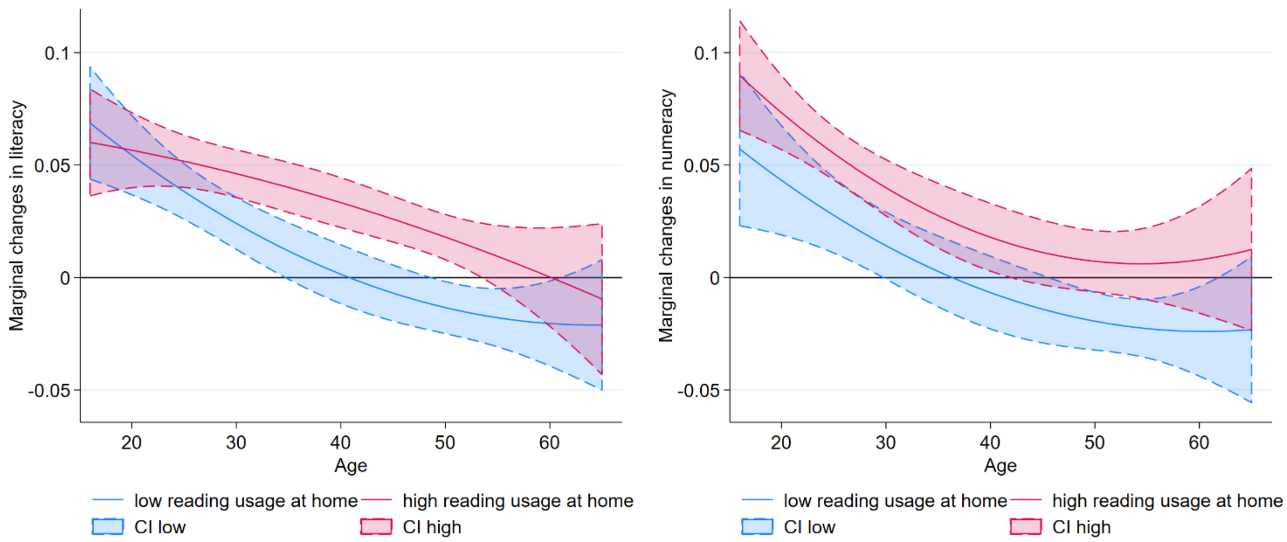

Fig. S7. Age-skill profiles by skill usage at home. Panel A: cumulative depiction of the predicted marginal change in skills at each age. Panel B: quadratic fit (with 95 percent confidence interval) of marginal annualized change in skills between the two waves by age, adjusted for reversion to the mean. Skills measured in SD units. Sample split by median of skill usage at home. Sample: employed workers, ages 16-65, weighted by sampling weights (N = 2,497). Data source: PIAAC-L.

A Cumulative age-skill profiles

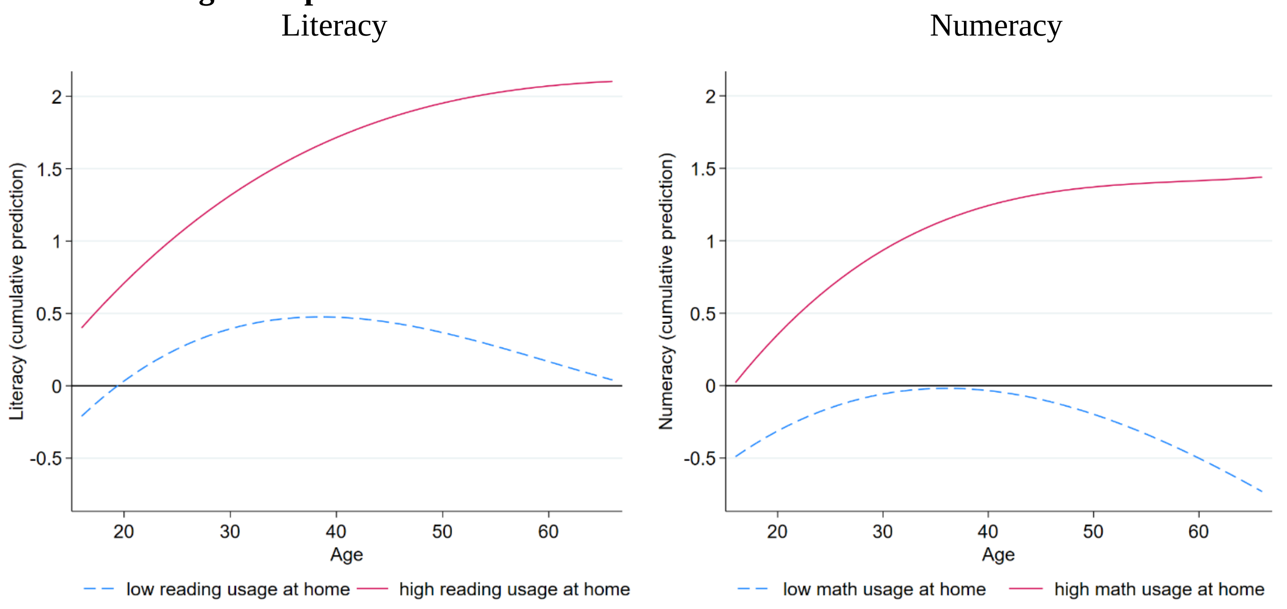

B Marginal skill changes by age

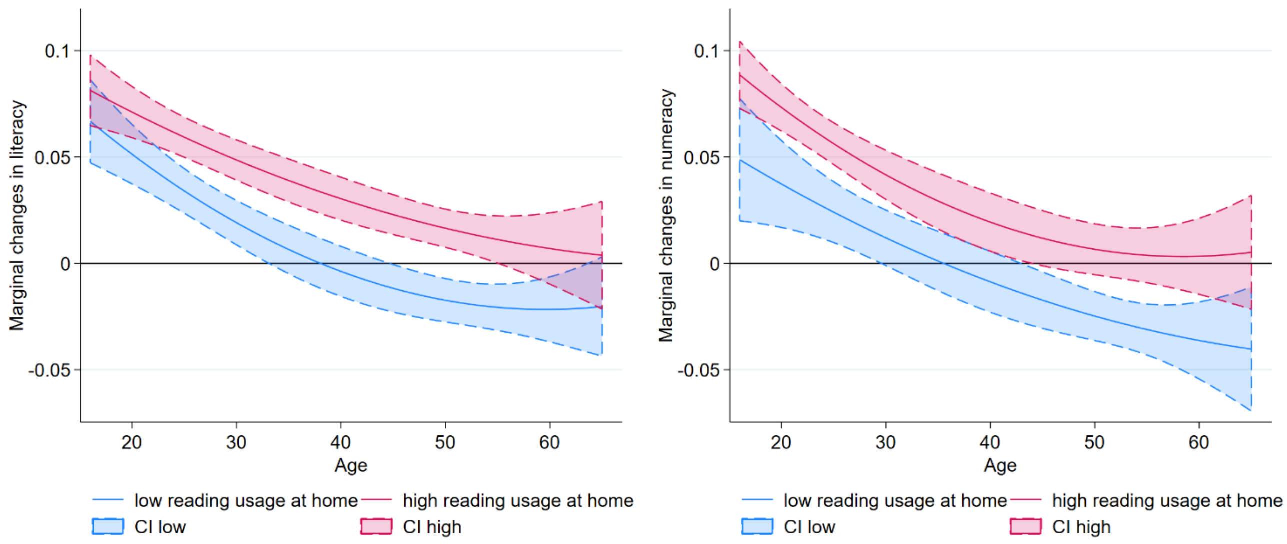

Fig. S8. Age-skill profiles by skill usage at home: Full population. Panel A: cumulative depiction of the predicted marginal change in skills at each age. Panel B: quadratic fit (with 95 percent confidence interval) of marginal annualized change in skills between the two waves by age, adjusted for reversion to the mean. Skills measured in SD units. Sample split by median of skill usage at home. Sample: full population, ages 16-65, weighted by sampling weights (N = 3,263). Data source: PIAAC-L.

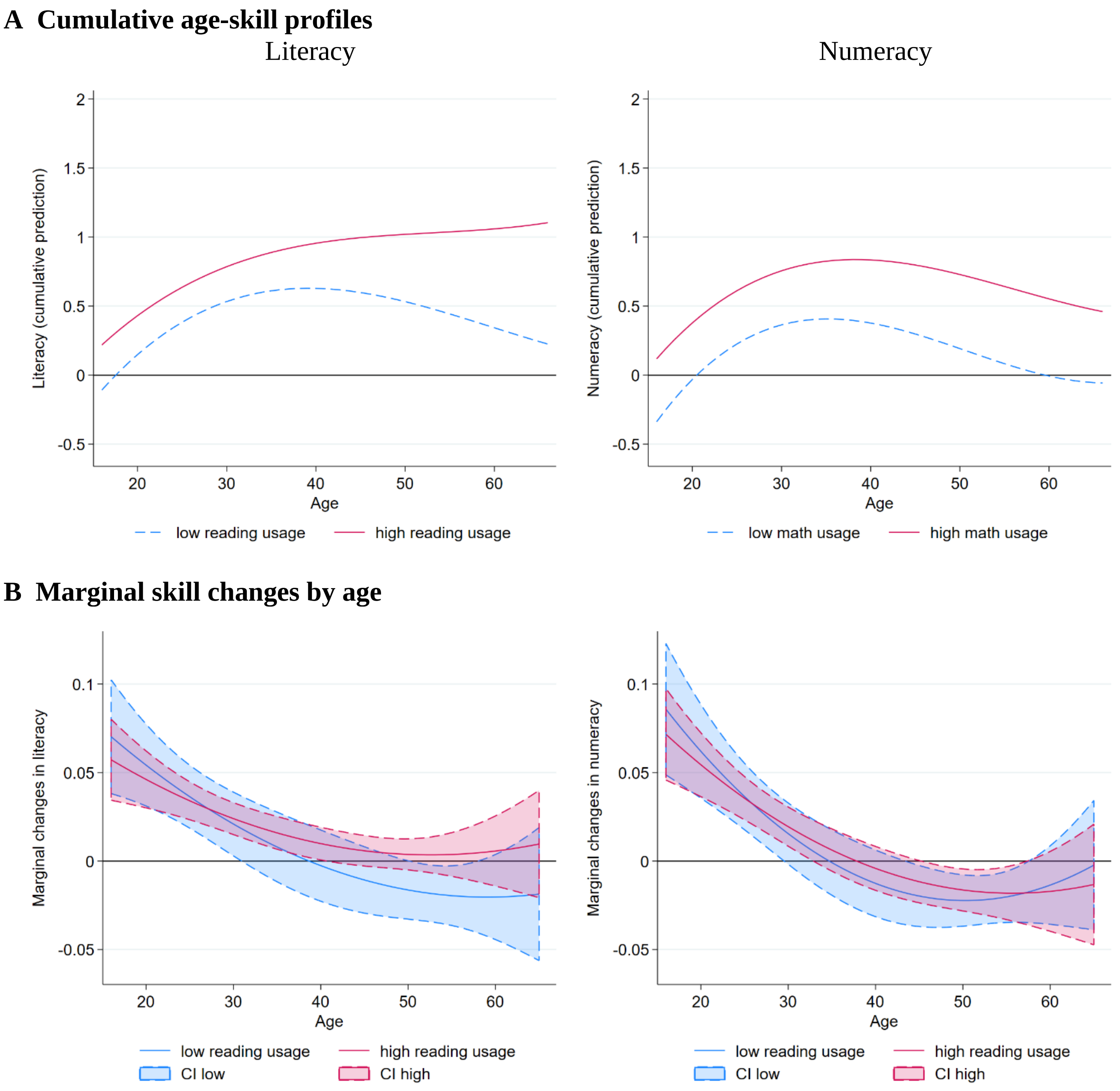

Fig. S9. Age-skill profiles by skill usage: Without adjustment for reversion to the mean. Raw scores not adjusted for reversion to the mean. Panel A: cumulative depiction of the predicted marginal change in skills at each age. Panel B: quadratic fit (with 95 percent confidence interval) of marginal annualized change in skills between the two waves by age. Skills measured in SD units. Sample split by median of skill usage at work and at home. Sample: employed workers, ages 16-65, weighted by sampling weights (N = 2,497). Data source: PIAAC-L.

A Cumulative age-skill profiles

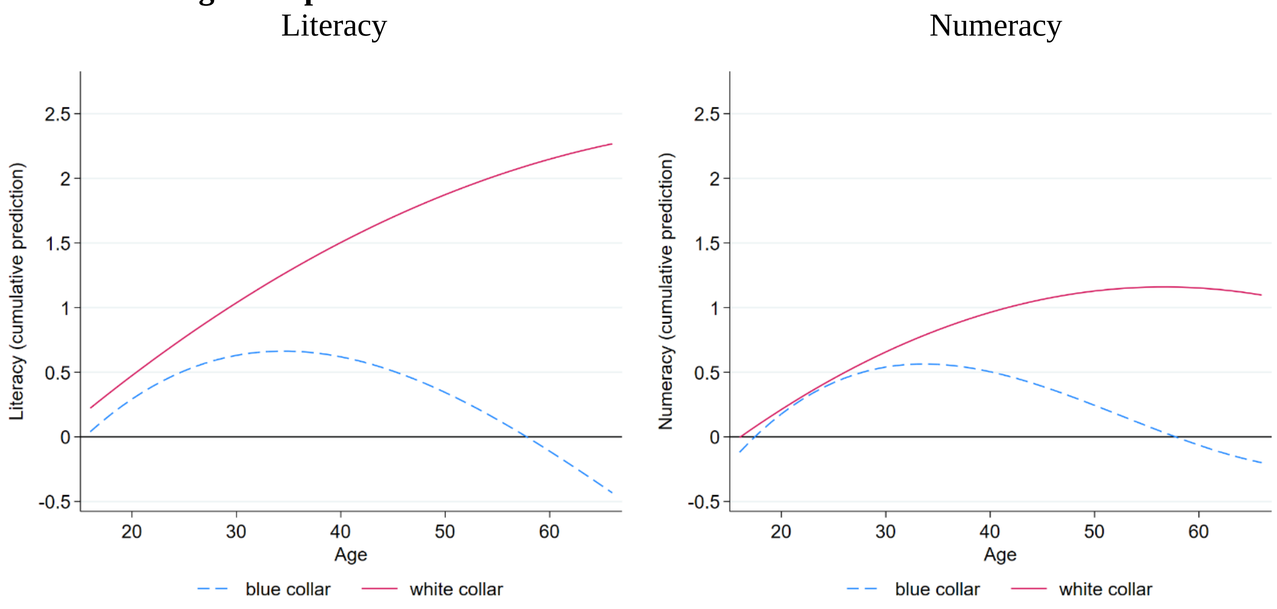

B Marginal skill changes by age

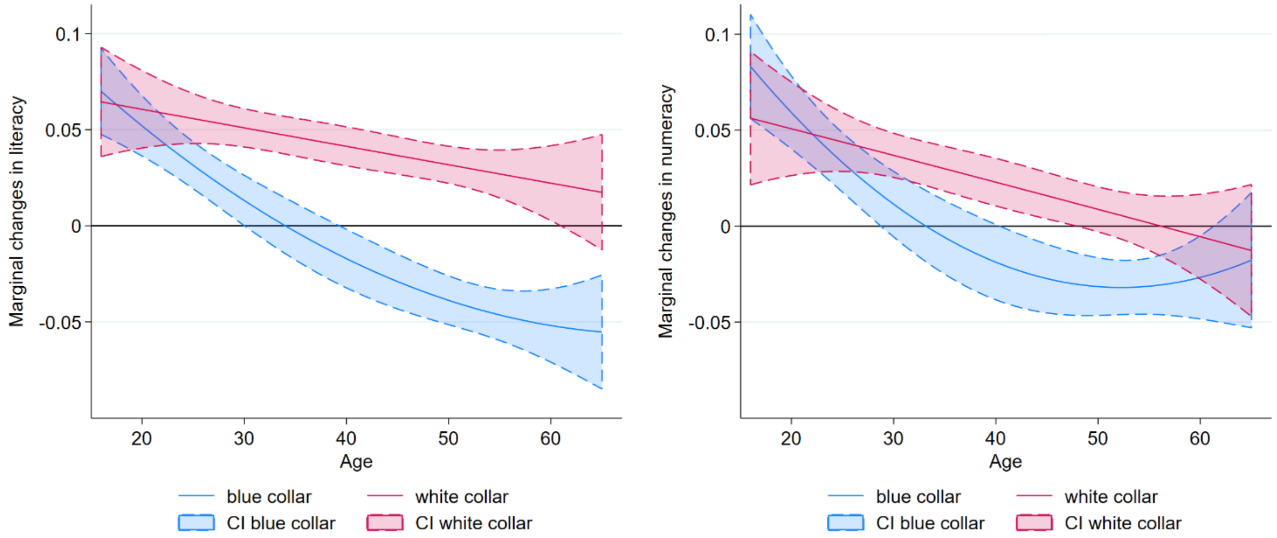

Fig. S10. Age-skill profiles by occupation. Panel A: cumulative depiction of the predicted marginal change in skills at each age. Panel B: quadratic fit (with 95 percent confidence interval) of marginal annualized change in skills between the two waves by age, adjusted for reversion to the mean. Skills measured in SD units. Sample split between blue- and white-collar workers. Sample: employed workers, ages 16-65, weighted by sampling weights (N = 2,497). Data source: PIAAC-L.

A Cumulative age-skill profiles

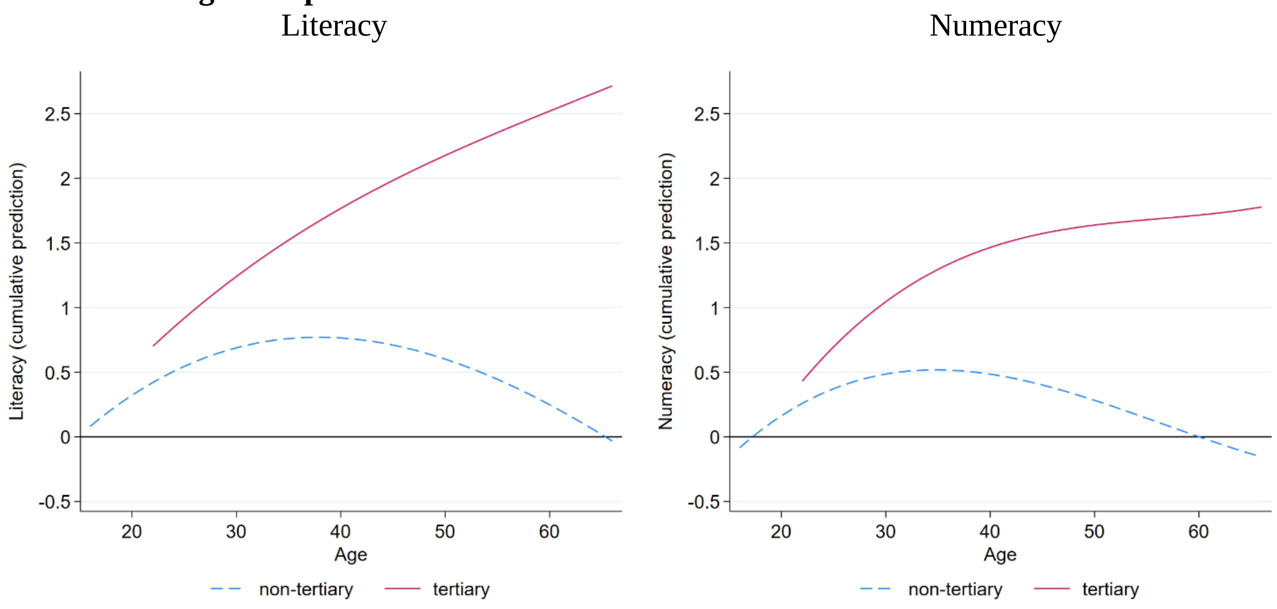

B Marginal skill changes by age

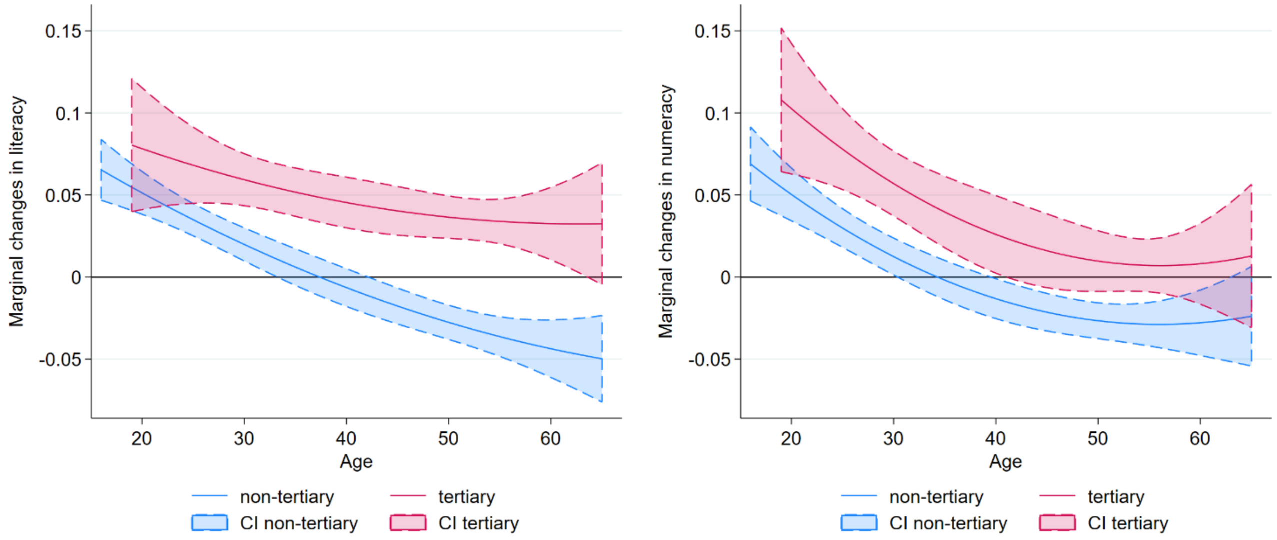

Fig. S11. Age-skill profiles by education. Panel A: cumulative depiction of the predicted marginal change in skills at each age. Panel B: quadratic fit (with 95 percent confidence interval) of marginal annualized change in skills between the two waves by age, adjusted for reversion to the mean. Skills measured in SD units. Sample split between workers with and without a tertiary education. Sample: employed workers, ages 16-65, weighted by sampling weights (N = 2,497). Data source: PIAAC-L.

A Cumulative age-skill profiles

Literacy   Numeracy

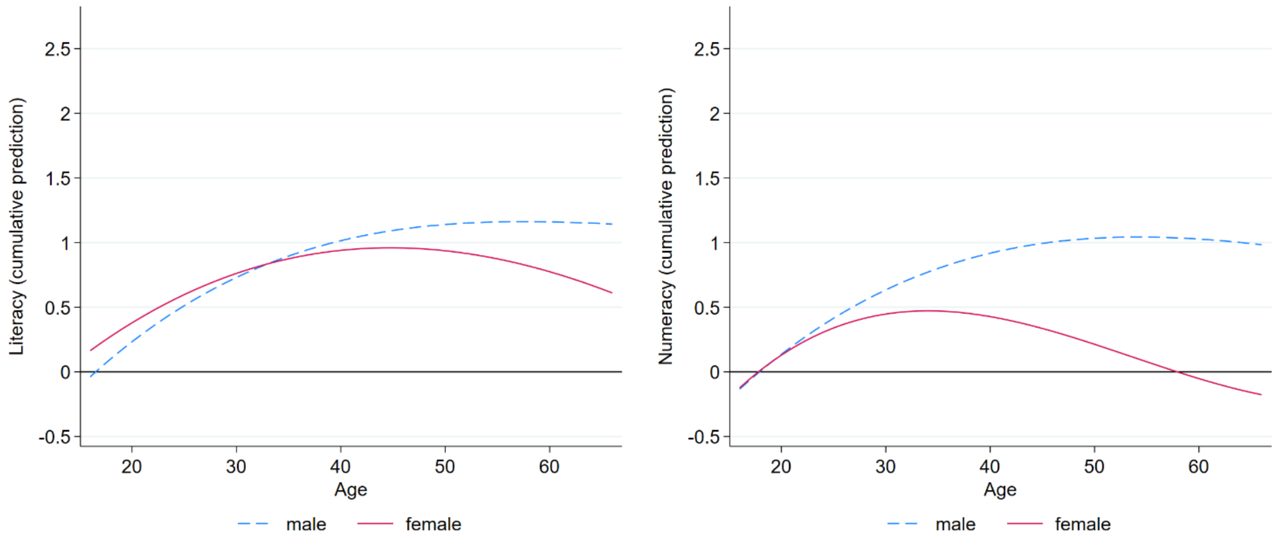

B Marginal skill changes by age

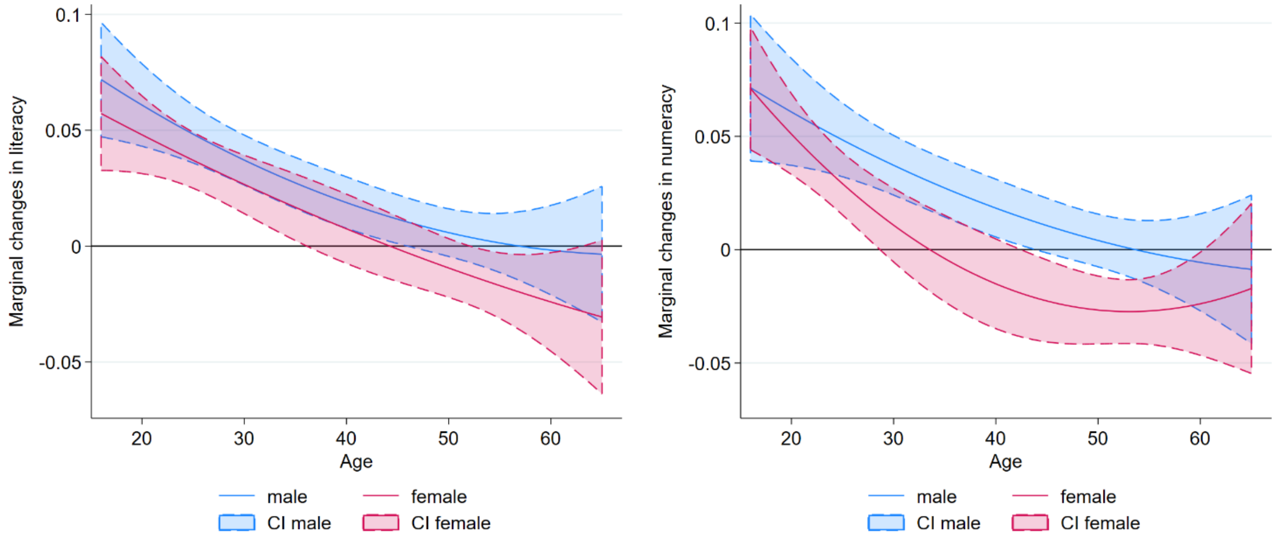

Fig. S12. Age-skill profiles by gender. Panel A: cumulative depiction of the predicted marginal change in skills at each age. Panel B: quadratic fit (with 95 percent confidence interval) of marginal annualized change in skills between the two waves by age, adjusted for reversion to the mean. Skills measured in SD units. Sample split by gender. Sample: employed workers, ages 16-65, weighted by sampling weights (N = 2,497). Data source: PIAAC-L.

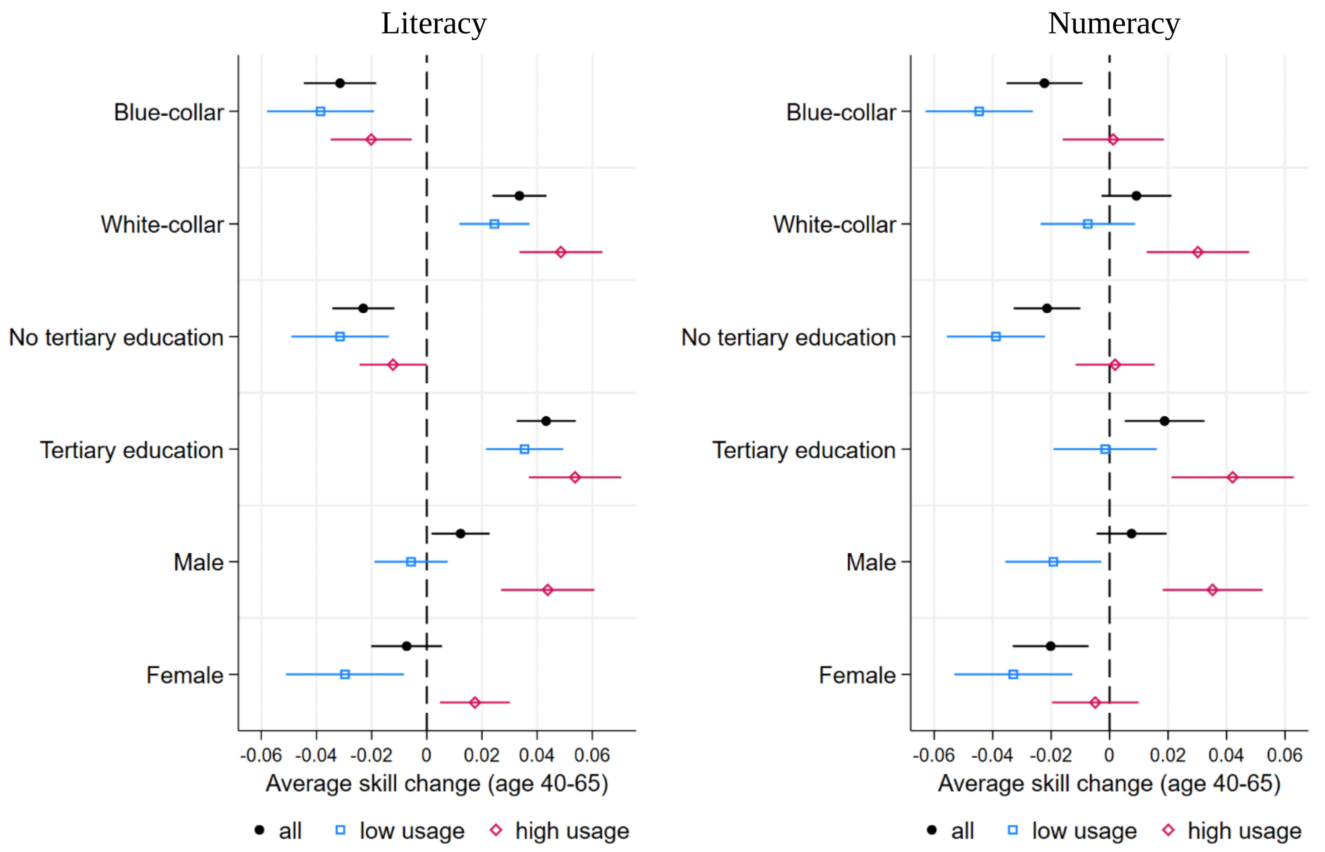

Fig. S13. Skill changes after age 40: Splits by medians of usage within subgroups. Average individual marginal annualized change in skills between the two waves, adjusted for reversion to the mean, and 95 percent confidence band. Skills measured in SD units. Positive values indicate increasing skills, negative values indicate decreasing skills. Subgroup means by blue-/white-collar occupations, (no) tertiary education, and gender, respectively. Low/high skill usage: below/above median of skill usage at work and at home within the respective subgroup. Sample: employed workers, ages 40-65, weighted by sampling weights. Data source: PIAAC-L.

A Cumulative age-skill profiles

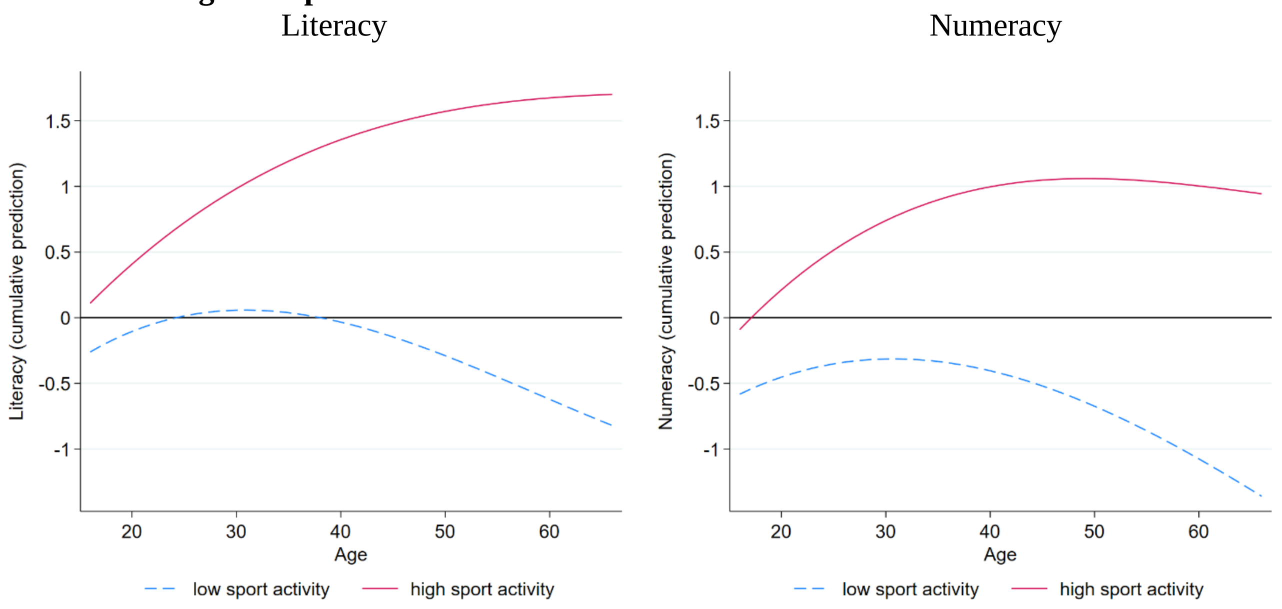

B Marginal skill changes by age

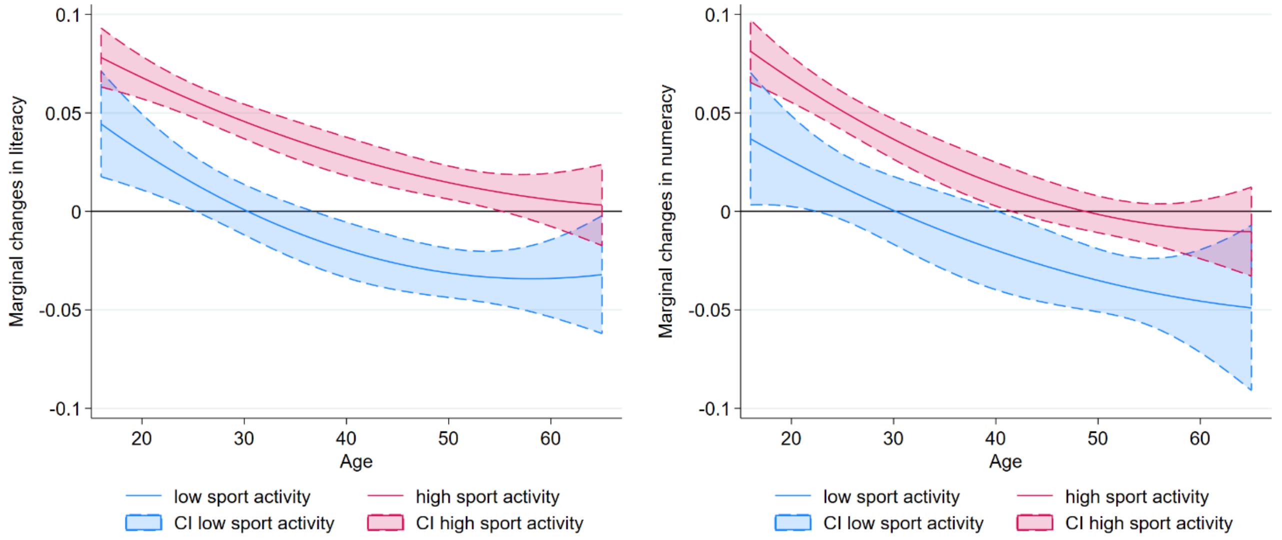

Fig. S14. Age-skill profiles by sports activity. Panel A: cumulative depiction of the predicted marginal change in skills at each age. Panel B: quadratic fit (with 95 percent confidence interval) of marginal annualized change in skills between the two waves by age, adjusted for reversion to the mean. Skills measured in SD units. Sample split by (not) doing any sports within the last three months in 2015. Sample: full population, ages 16-65, weighted by sampling weights (N = 3,262). Data source: PIAAC-L.

A Cumulative age-skill profiles

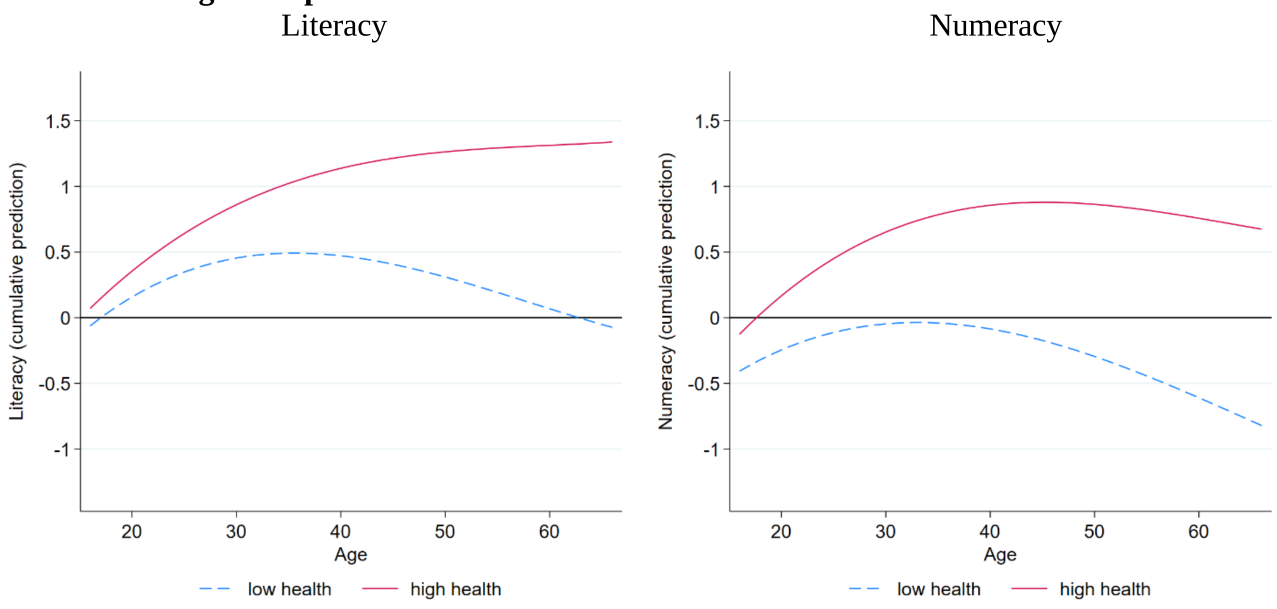

B Marginal skill changes by age

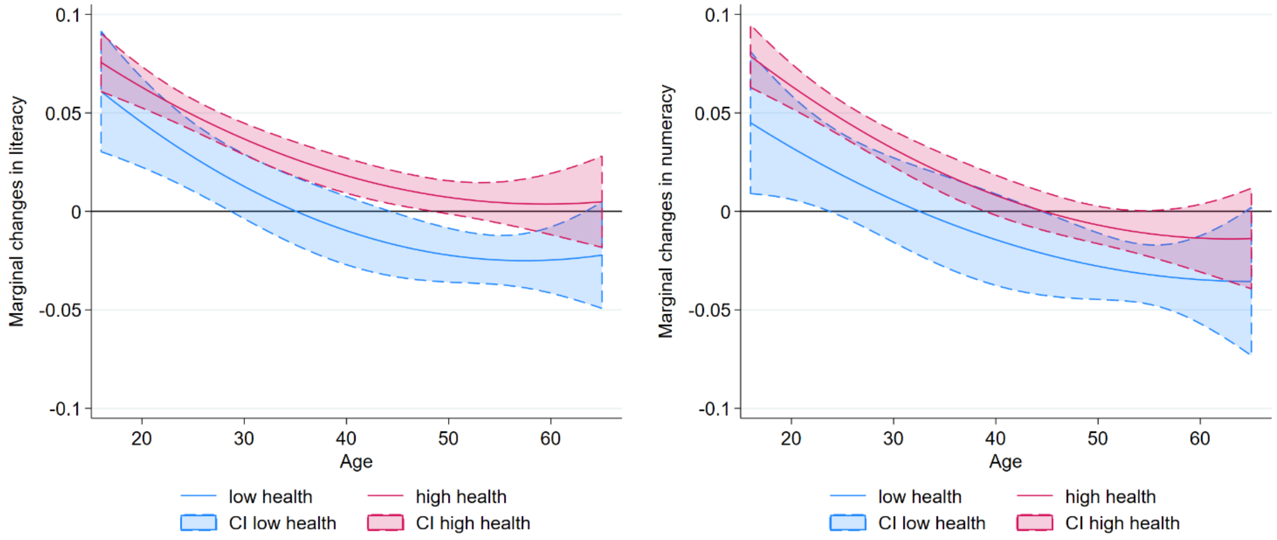

Fig. S15. Age-skill profiles by health status. Panel A: cumulative depiction of the predicted marginal change in skills at each age. Panel B: quadratic fit (with 95 percent confidence interval) of marginal annualized change in skills between the two waves by age, adjusted for reversion to the mean. Skills measured in SD units. Sample split by low (poor, less good, or satisfactory) vs. high (good or very good) health status. Sample: full population, ages 16-65, weighted by sampling weights (N = 3,262). Data source: PIAAC-L.

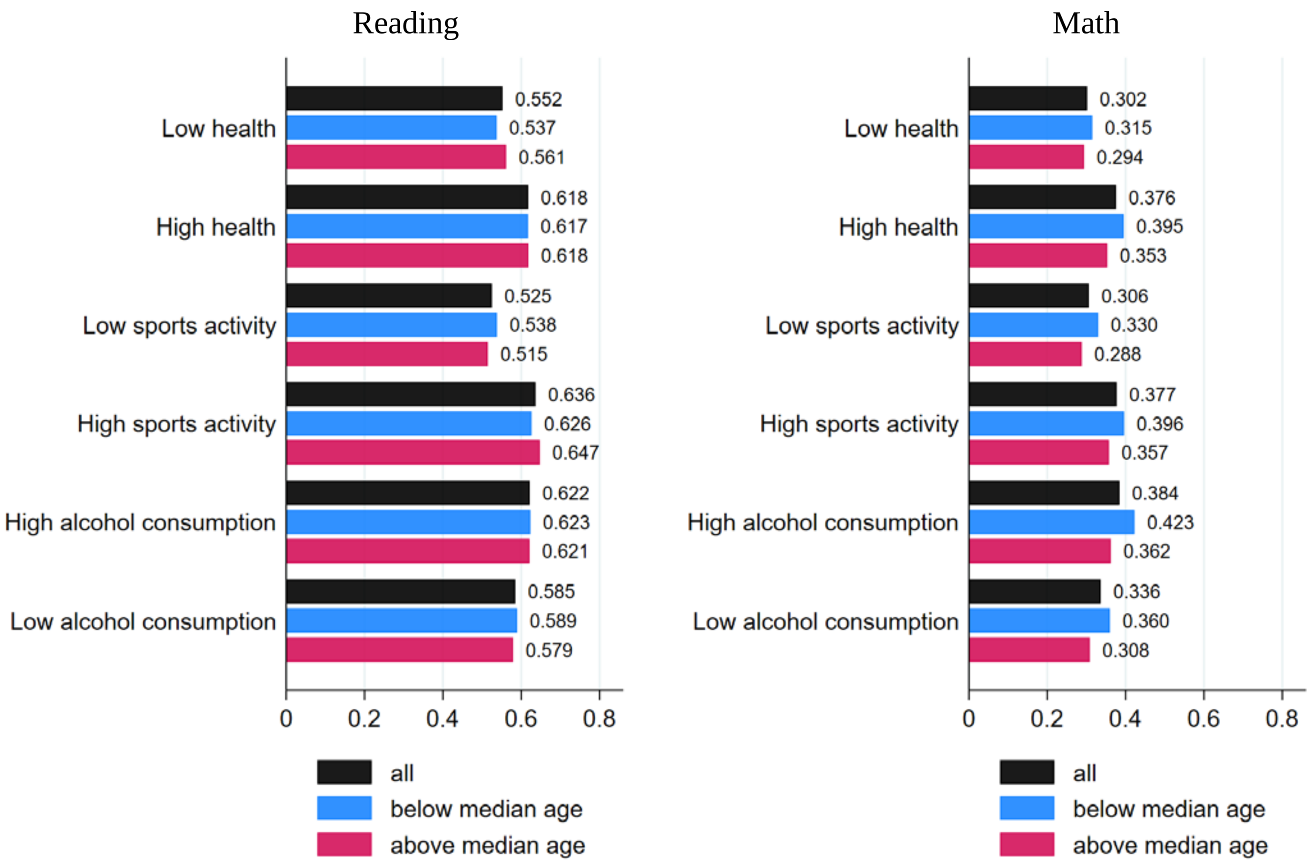

Fig. S16. Skill usage: By physical/health background and age. Average of indicators of at least monthly skill usage in different categories at work and at home. Subgroup means by low/high health, low/high sports activity, and high/low alcohol consumption, respectively. Below/above median age: sample split by median of age (43). Sample: employed workers, ages 16-65, weighted by sampling weights. Data source: PIAAC-L.

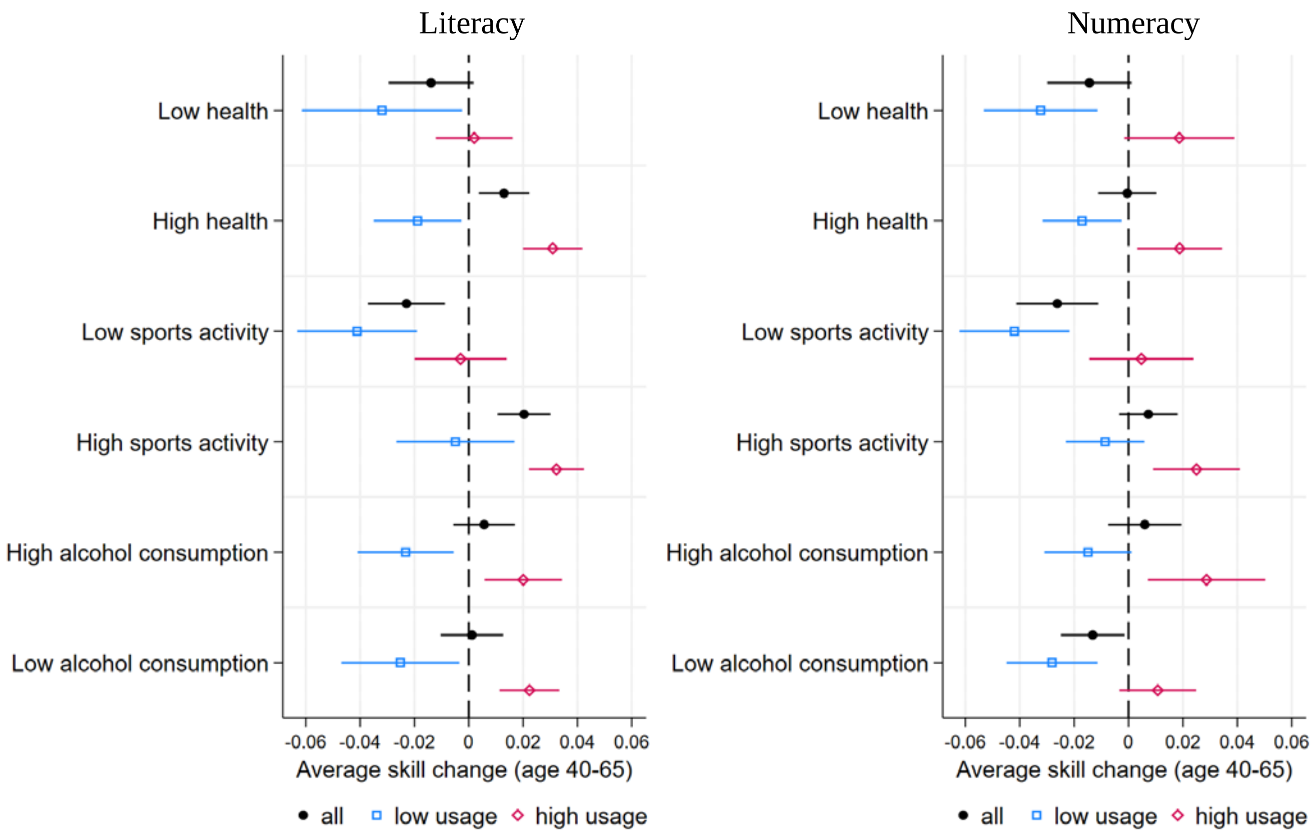

Fig. S17. Skill changes after age 40: By physical/health background and skill usage. Average individual marginal annualized change in skills between the two waves, adjusted for reversion to the mean, and 95 percent confidence band. Skills measured in SD units. Positive values indicate increasing skills, negative values indicate decreasing skills. Subgroup means by low/high health, low/high sports activity, and high/low alcohol consumption, respectively. Low/high skill usage: below/above median of skill usage at work and at home. Sample: employed workers, ages 40-65, weighted by sampling weights. Data source: PIAAC-L.

A Cumulative age-skill profiles

Literacy Numeracy

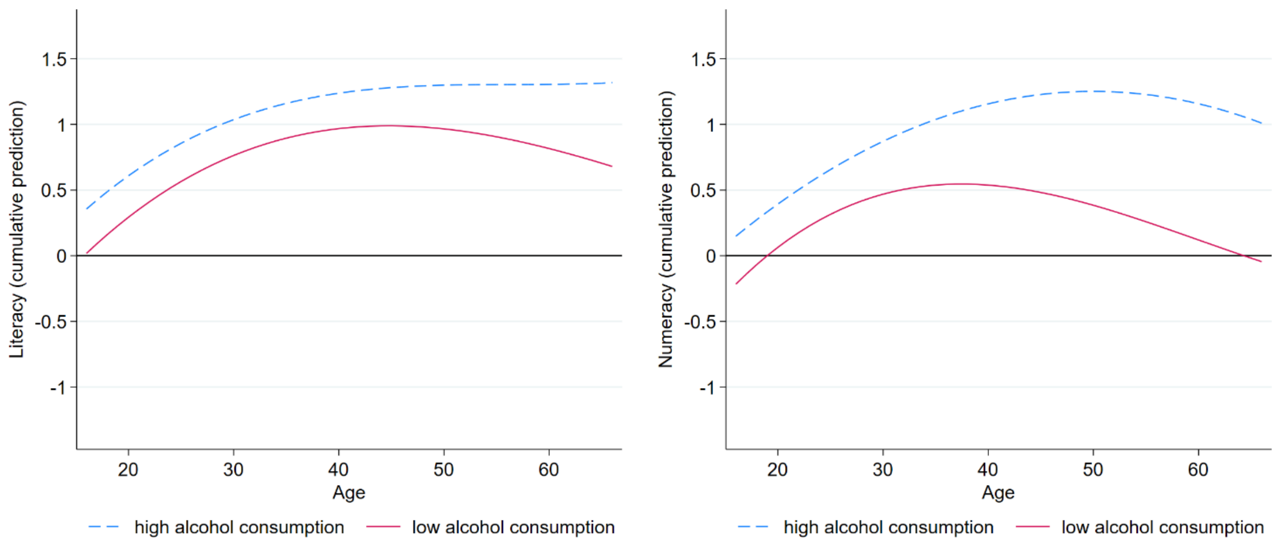

B Marginal skill changes by age

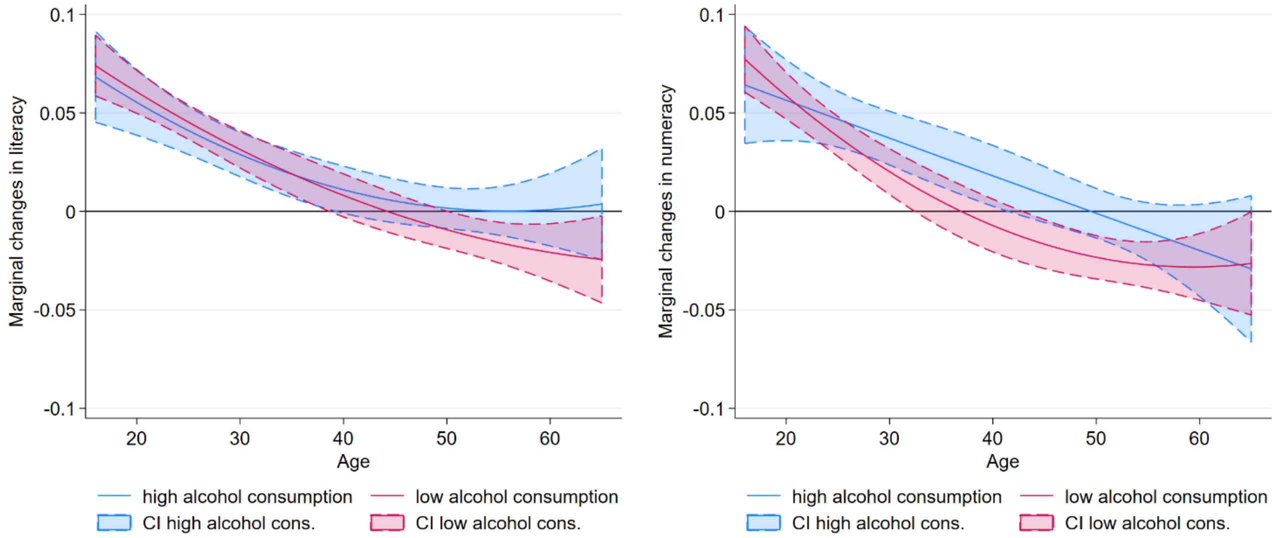

Fig. S18. Age-skill profiles by alcohol consumption. Panel A: cumulative depiction of the predicted marginal change in skills at each age. Panel B: quadratic fit (with 95 percent confidence interval) of marginal annualized change in skills between the two waves by age, adjusted for reversion to the mean. Skills measured in SD units. Sample split by high (2 to 3 times a week or 4 times a week or more) vs. low (never, once a month or less, or 2 to 4 times a month) alcohol consumption. Sample: full population, ages 16-65, weighted by sampling weights (N = 3,262). Data source: PIAAC-L.

A Using original weights

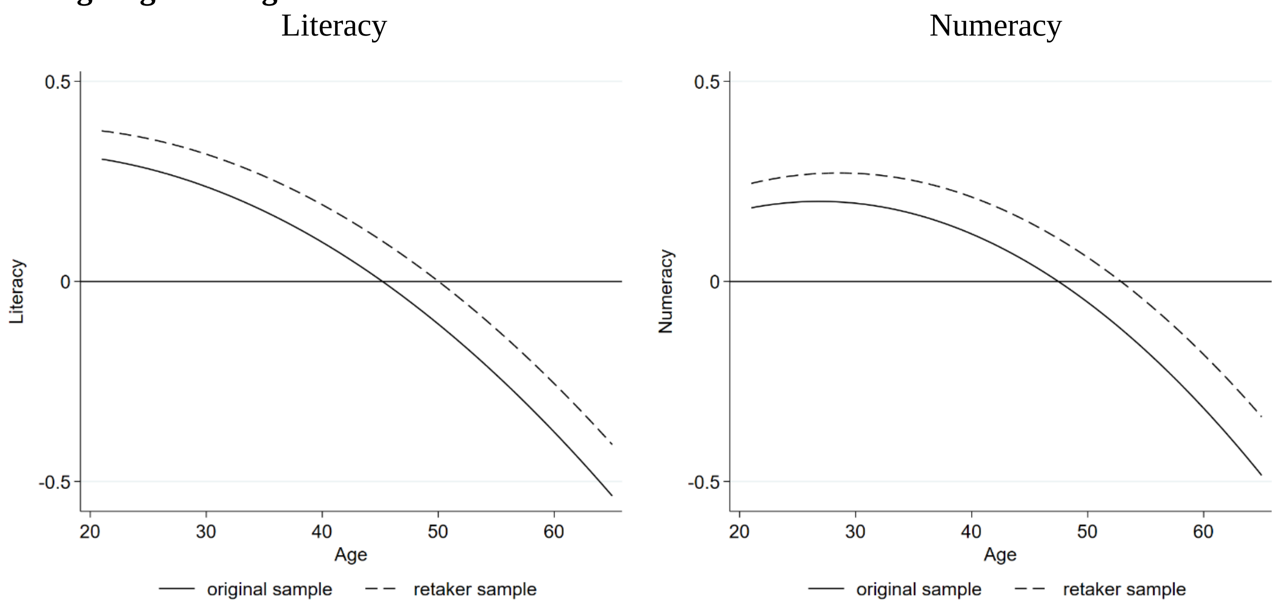

B Using new sampling weights for retaker sample

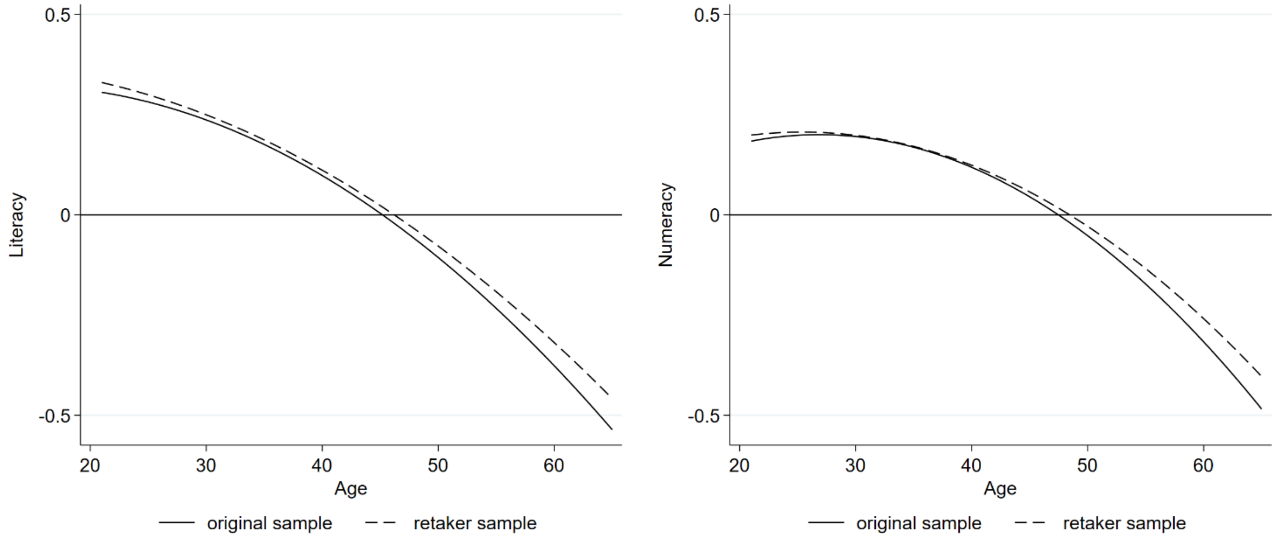

Fig. S19. Cross-sectional age-skill profiles: Original vs. retaker sample. Quadratic fit of the cross-sectional association between age and skills in the initial (2012) wave, estimated over 21-65 age range. Panel A: original and retaker sample using original sampling weights. Panel B: original and retaker sample using new PIAAC-L sampling weights for retaker sample. Skills measured in SD units. Original IRT scaling. Sample: full population, ages 16-65, weighted by sampling weights. Original sample: N= 5,379. Retaker sample: N = 3,263. Data source: PIAAC-L.

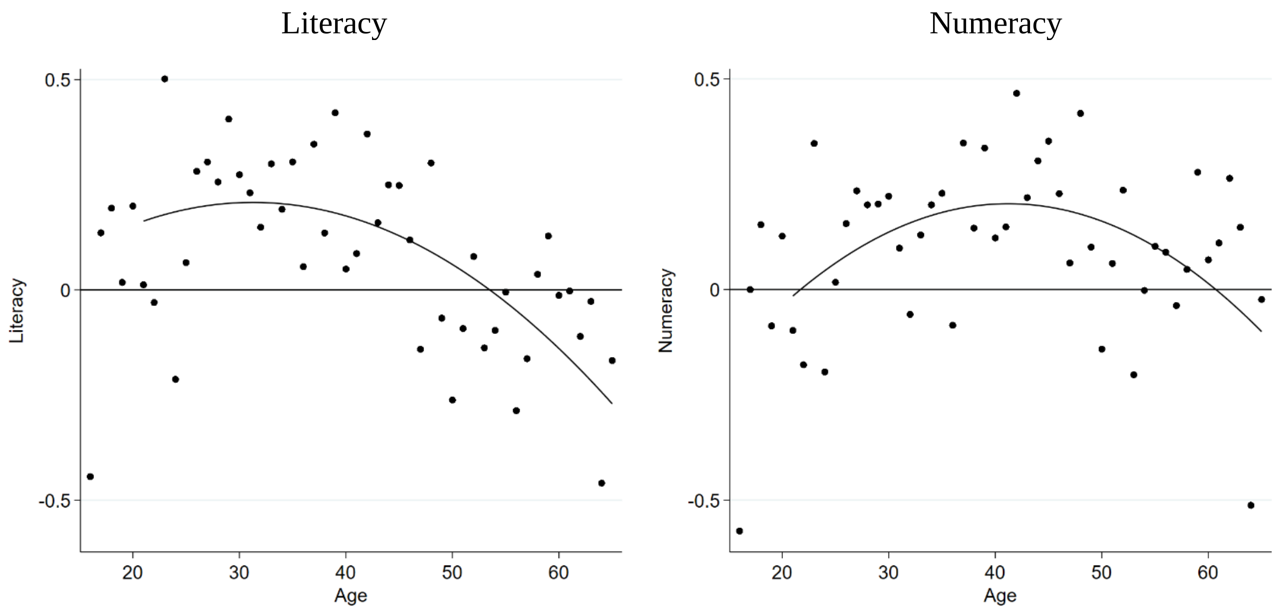

Fig. S20. Cross-sectional age-skill profiles: Employed sample. Cross-sectional association between age and skills in the initial (2012) wave. Dots: average skills by age. Line: quadratic fit (estimated over 21-65 age range). Skills measured in SD units. Sample: employed workers, ages 16-65, weighted by sampling weights (N = 2,497). Data source: PIAAC-L.

Table S1. Heterogeneity in marginal changes in skills: Different measures of skill usage. Least squares regressions weighted by sampling weights. Dependent variable: individual marginal annualized change in skills between the two waves, adjusted for reversion to the mean. Panel A: literacy skills. Panel B: numeracy skills. Skills measured in SD units. Skill usage measures refer to average of indicators of at least monthly skill usage in different categories, except for "skill usage (full range)", which uses the underlying five-point scale of usage (ranging from never to every day), linearized from zero to one. Background controls: white-collar occupation, tertiary education, and female. Sample: employed workers, ages 16-65. Regressions use ten plausible values of skill measurement per observation (individual). Standard errors clustered at the individual level in parentheses. Significance level: *** 1 percent, ** 5 percent, * 10 percent. Data source: PIAAC-L.

A Literacy

	(1)	(2)	(3)	(4)	(5)	(6)
Skill usage at work	0.079***		0.067***			
	(0.012)		*(0.013)*			
Skill usage at home		0.071***	0.036***			
		(0.012)	*(0.012)*			
Reading skill usage				0.097***		
				(0.016)		
Math skill usage				0.022		
				(0.014)		
Skill usage (full range)					0.145***	0.078***
					(0.020)	*(0.023)*
Age and age squared	yes	yes	yes	yes	yes	yes
Background controls	no	no	no	no	no	yes
R^2 (adj.)	0.044	0.031	0.047	0.047	0.049	0.071
Observations	2,497	2,497	2,497	2,497	2,497	2,497

B Numeracy

	(1)	(2)	(3)	(4)	(5)	(6)
Skill usage at work	0.073***		0.064***			
	(0.013)		*(0.012)*			
Skill usage at home		0.058***	0.030**			
		(0.015)	*(0.014)*			
Reading skill usage				0.082***		
				(0.018)		
Math skill usage				0.058***		
				(0.017)		
Skill usage (full range)					0.128***	0.096***
					(0.023)	*(0.024)*
Age and age squared	yes	yes	yes	yes	yes	yes
Background controls	no	no	no	no	no	yes
R^2 (adj.)	0.036	0.025	0.037	0.046	0.038	0.046
Observations	2,497	2,497	2,497	2,497	2,497	2,497

Table S2. Heterogeneity in marginal changes in skills: By physical/health background and skill usage. Least squares regressions weighted by sampling weights. Dependent variable: individual marginal annualized change in skills between the two waves, adjusted for reversion to the mean. Panel A: literacy skills. Panel B: numeracy skills. Skills measured in SD units. Skill usage: average of indicators of at least monthly skill usage in different categories at work and at home. Sample: employed workers, ages 16-65. Regressions use ten plausible values of skill measurement per observation (individual). Standard errors clustered at the individual level in parentheses. Significance level: *** 1 percent, ** 5 percent, * 10 percent. Data source: PIAAC-L.

A Literacy

	(1)	(2)	(3)	(4)	(5)	(6)
Skill usage	0.108***				0.089***	0.045***
	(0.015)				*(0.014)*	*(0.015)*
High health		0.023***			0.014*	0.011
		(0.008)			*(0.007)*	*(0.007)*
High sports activity			0.043***		0.032***	0.026***
			(0.007)		*(0.007)*	*(0.007)*
Low alcohol consumption				-0.003	0.002	0.008
				(0.006)	*(0.006)*	*(0.006)*
White-collar occupation						0.030***
						(0.007)
Tertiary education						0.028***
						(0.008)
Female						-0.019***
						(0.006)
Age and age squared	yes	yes	yes	yes	yes	yes
R^2 (adj.)	0.046	0.023	0.036	0.018	0.056	0.077
Observations	2,497	2,497	2,496	2,496	2,495	2,495

B Numeracy

	(1)	(2)	(3)	(4)	(5)	(6)
Skill usage	0.100***				0.086***	0.068***
	(0.018)				*(0.016)*	*(0.017)*
High health		0.021**			0.011	0.009
		(0.010)			*(0.009)*	*(0.009)*
High sports activity			0.038***		0.031***	0.028***
			(0.009)		*(0.008)*	*(0.008)*
Low alcohol consumption				-0.019**	-0.013*	-0.007
				(0.007)	*(0.007)*	*(0.007)*
White-collar occupation						0.006
						(0.009)
Tertiary education						0.020*
						(0.012)
Female						-0.021***
						(0.008)
Age and age squared	yes	yes	yes	yes	yes	yes
R^2 (adj.)	0.036	0.020	0.028	0.020	0.046	0.053
Observations	2,497	2,497	2,496	2,496	2,495	2,495

Table S3. Descriptive statistics. Variables refer to initial (2012) wave unless noted otherwise. Skills measured in SD units. Sample: full population and employed workers, respectively, ages 16-65, weighted by sampling weights. Data source: PIAAC-L.

	Full sample		Employed sample	
	Mean	Std. dev.	Mean	Std. dev.
	(1)	(2)	(3)	(4)
Age	41.329	13.625	41.869	12.259
White-collar occupation	0.378		0.501	
Tertiary education	0.306		0.353	
Female	0.499		0.471	
Literacy (2012)	0	1	0.094	0.962
Literacy (2015)	0.049	1.017	0.126	0.976
Change in literacy (annual)	0.014	0.155	0.013	0.153
Numeracy (2012)	0	1	0.125	0.943
Numeracy (2015)	0.020	1.042	0.123	0.996
Change in numeracy (annual)	0.006	0.174	0.006	0.172
Observations	3,263		2,497	
